# Antimalarial Activity of Plant Metabolites

**DOI:** 10.3390/ijms19051382

**Published:** 2018-05-06

**Authors:** Wen-Hui Pan, Xin-Ya Xu, Ni Shi, Siu Wai Tsang, Hong-Jie Zhang

**Affiliations:** 1 School of Chinese Medicine, Hong Kong Baptist University, 7 Baptist University Road, Kowloon Tong, Kowloon, Hong Kong SAR, China; 13480448@life.hkbu.edu.hk (W.-H.P.); xuxinya@scsio.ac.cn (X.-Y.X.); 14252252@life.hkbu.edu.hk (N.S.); tsang@hkbu.edu.hk (S.W.T.);; 2 CAS Key Laboratory of Tropical Marine Bio-resources and Ecology, Guangdong Provincial Key Laboratory of Applied Marine Biology, South China Sea Institute of Oceanology, Chinese Academy of Science, Guangzhou 510070, China

**Keywords:** anti-malaria activity, plants, natural products, ethnopharmacology, *Plasmodium* parasites

## Abstract

Malaria, as a major global health problem, continues to affect a large number of people each year, especially those in developing countries. Effective drug discovery is still one of the main efforts to control malaria. As natural products are still considered as a key source for discovery and development of therapeutic agents, we have evaluated more than 2000 plant extracts against *Plasmodium falciparum*. As a result, we discovered dozens of plant leads that displayed antimalarial activity. Our phytochemical study of some of these plant extracts led to the identification of several potent antimalarial compounds. The prior comprehensive review article entitled “Antimalarial activity of plant metabolites” by Schwikkard and Van Heerden (2002) reported structures of plant-derived compounds with antiplasmodial activity and covered literature up to the year 2000. As a continuation of this effort, the present review covers the antimalarial compounds isolated from plants, including marine plants, reported in the literature from 2001 to the end of 2017. During the span of the last 17 years, 175 antiplasmodial compounds were discovered from plants. These active compounds are organized in our review article according to their plant families. In addition, we also include ethnobotanical information of the antimalarial plants discussed.

## 1. Introduction

Malaria is still considered as a major global health problem, affecting a large population of the world. According to World Health Organization (WHO), there were about 216 million malaria cases globally and 445,000 deaths in 2016. Most of the cases and the deaths occurred in the WHO African region and affected primarily children and pregnant women [1].

*P. falciparum*, *P. vivax*, *P. ovale*, *P. malariae* and *P. knowlesi* are the five *Plasmodium* species that cause malaia disease in humans. *P. falciparum* is the deadliest strain that causes malaria and this form of parasite predominates in Africa [2,3]. Humans get infected with malaria parasites through the bites of female anopheline mosquitoes [4]. The *Plasmodium* parasites travel through blood and become mature and reproduce in the liver, leading to malaria disease. The common symptoms of malaria are fever and headache, and in severe cases, malaria causes death [5].

Currently, there is no commercially available malaria vaccine, though efforts to develop vaccines are still ongoing. The most promising vaccine candidate is RTS, S/AS01, which is in clinical trials for treatment of malaria caused by *P. falciparum* [1]. Several medications are available to prevent malaria for travellers in malaria-endemic countries, and a number of drugs are available for treatment of those who have the disease [6].

**Figure 1:**
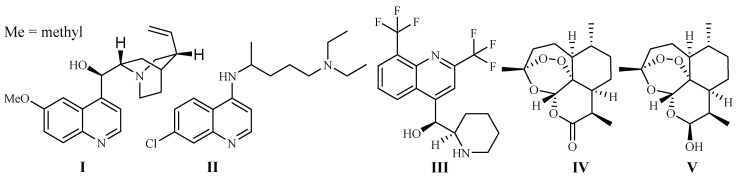
Antimalarial drugs developed from plants.

In 1820, French scientists Pelletier and Caventou discovered quinine (**I**) as the first antimalarial drug, which was originally isolated from the barks of *Cinchona* species (Rubiaceae) (Figure 1). *Cinchona* plants are used as folk medicines in South America by Peruvian Indians, and they were introduced to Europe in the 1700s [7]. Quinine is commercially obtained by solvent extraction from wild-growing *Cinchona* species in South America, or the plants cultivated in Indonesia [8].

Chloroquine (**II**) and its derivative 4-aminoquinoline were developed in the 1940s. They are widely used as antimalarial drugs, even today. The effectiveness of the drugs, however, has declined rapidly since the 1960s, which was due to the development of drug resistance by *P. falciparum* strains, leading to a significant malaria-associated death rate [9]. Mefloquine (**III**), is a 4-quinolinemethanol derivative obtained via total synthesis. It was introduced as a new antimalarial drug in 1985. The drug can be used to treat mild or moderate malaria but should not be used to treat severe malaria [10].

The current antimalarial drug of choice is artemisinin (Qinghaosu, **IV**), which was originally obtained from the leaves of Qinghao [*Artemisia annua* L. (Asteraceae)] in the 1970s. The compound is clinically effective against chloroquine-resistant malaria strains [11]. The plant Qinghao has been used as a traditional medicine in China for the treatment of fever of malaria origin for about 2000 years [12]. A large number of artemisinin analogs have also been synthesized. The best known among these derivatives are artemether, arteether (artemotil), artesunate and artenimol (β-dihydroartemisinin, DHA) [13]. Artemisinin and its semi-synthetic derivatives have shown better efficacy than quinine for both children and adults patients [14].

Although the anti-parasitic mechanism of action of artemisinin is still in question [15], the endoperoxide bridge is regarded as the key functional group responsible for eliciting free radical-mediated parasite killing mechanisms. According to one school of thought, *Plasmodium* parasites live and reproduce in the host by ingesting red blood cell hemoglobin. This results in an accumulation of heme Fe^2+^ in the parasite. Fe^2+^ firstly interacts and cleaves the peroxide bridge of artemisinin to form highly reactive free radicals, which in turn cause a series of parasite molecular events and eventually kill the parasites [16]. The most used artemisinin derivative today is the prodrug, dihydroartemisinin (**V**), which is metabolized into the pharmacologically active artimisinin (**IV**) in the body [17]. Artesunate was investigated as a potential inhibitor of the essential *P. falciparum* exported protein 1 (EXP1), a membrane glutathione *S*-transferase [18].

Clinically, it is unwise to use artemisinin as the lone therapy due to the potential risk of the parasites to develop resistance to this drug. Indeed, artemisinin drug resistance has been already detected in some Southern Asian countries: Lao People’s Democratic Republic, Cambodia, Thailand, Myanmar and Viet Nam [1]. This risk has led to the withdrawal of artemisinin monotherapy from clinical applications.

At present, the use of artemisinins in combination with other drugs, known as artemisinin-based combination therapy (ACT), is the most effective to treat malarial disease caused by *P. falciparum* infection. Five currently available ACTs are artemether in combination with lumefantrine, and four other forms based on artesunate in combination with amodiaquine (two formulations), mefloquine and sulfadoxine+pyrimethamine [1]. Unfortunately, resistance has already been detected to both artemisinin and artesunate components of the multiple ACTs, as well as the non-artemisinin-based combination comprising atovaquone and proguanil. The current availbale antimalarial drugs are listed in Table 1 [1,19,20].

In the search for drug candidates, the initial step is the employment of appropriate bioassays to evaluate the antiplasmodial activity of a candidate. Several strains of *P. falciparum* have been used for this purpose in the past. The strains of *P. falciparum* that are sensitive and resistant to chloroquine are frequently used for antimalarial drug discovery programs. D6, D10, 3D7, TM4 and PoW are chloroquine-sensitive strains, whereas, W2, FCR-3, FcB1 and Dd2 represent chloroquine-resistant strains, and K1 is a multidrug resistant strain.

The need to discover effective and non-drug resistant antimalarial drugs is urgent as *Plasmodium* strains have already developed resistance to all of today’s available drugs including artemisinin. In that regard, it should be noted that natural products have proven to be a valuable source for the discovery of novel antimalarial therapeutic agents since the discovery of the first antimalarial drug in 1800s [20]. We, thus, pursued this approach in the search for new antimalarial potential drug leads.

**Figure 2:**
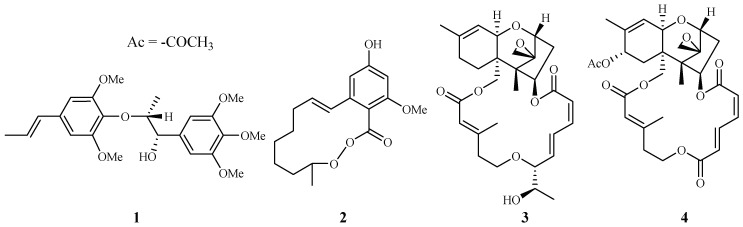
Compounds from *R. decursiva* and *F. fitulosa*.

In our antimalarial drug discovery program, we have evaluated more than 2000 plant extracts against D6 and W2 strains of *P. falciparum*. Dozens of these plants displayed antimalarial activity. Several of these plant leads were investigated further to uncover their antimalarial constituents. Phytochemical separation of these plant leads guided by bioassays led to the identification of ten new and 13 known active compounds [21]. Some of these compounds demonstrated potent antimalarial activity [22,23,24,25,26,27,28,29]. For example, polysyphorin (**1**) and rhaphidecurperoxin (**2**), isolated from *Rhaphidophora decursiva* (Araceae), showed antimalarial activities of 1.5 and 1.4 µM against the W2 clones of *P. falciparum*, respectively (Figure 2) [22]. Two trichothecenes, roridin E (**3**) from *R. decursiva* (Araceae) and verrucarin L acetate (**4**) from *Ficus fistulosa* (Moraceae), were found to potently inhibit the parasite growth with IC_50_ values in the sub-nano molar range [24].

De-replication to avoid duplication of previous efforts is an essential step in drug discovery protocols. To that end, we conducted a thorough review of the published literature on natural products possessing antimalarial activity. Previously, a literature review by Schwikkard and Van Heerden [30], covered plant-derived antiplasmodial active natural compounds up to the year 2000. The compounds were organized according to the origins of their corresponding plant families. The current review seeks to supplement the review of Schwikkard and Van Heerden. Compounds with antimalarial activity will also be organized according to their plant family of origin (Table 2). Literature published between 2001 and 2017 have been covered. In addition, we also included the ethnobotanic information of plants that have been used as folk medicines for the treatment of malarial disease (Table 3).

## 2. Plant-derived Antimalarial Compounds

### 2.1. Annonaceae–Asteraceae Families

#### 2.1.1. Annonaceae Family

**Figure 3:**
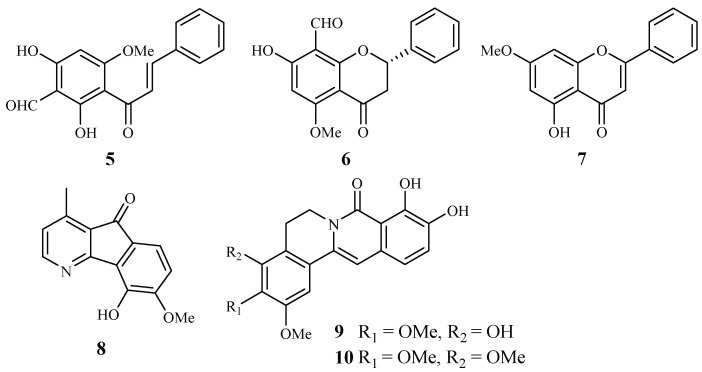
Compounds from Annonaceae plants.

Annonaceae is a family of flowering plants consisting of about 2400 species. Two plants in this family have been phytochemically investigated for their antiplasmodial and cytotoxic activities. From the leaves of *Friesodielsia discolor*, Prawat et al. isolated two new flavonoids, 3′-formyl-2′,4′-dihydroxy-6′-methoxychalcone (**5**), 8-formyl-7-hydroxy-5-methoxyflava-none (**6**), and the known tectochrysin (**7**) (Figure 3) [31]. They displayed antiplasmodial activity against the K1 multidrug resistant strain of *P. falciparum* with IC_50_ values of 9.2, 9.3 and 7.8 μM, respectively. However, these compounds also exhibited cytotoxicity against the cancer cell lines KB and MCF-7, with the IC_50_ values ranging from 13.9–34.5 μM.

According to Mueller et al. [32], 5-hydroxy-6-methoxyonychine (**8**), an alkaloid obtained from the roots of the Australian tree plant *Mitrephora diversifolia*, showed IC_50_ values of 9.9 and 11.4 μM against the 3D7 and Dd2 clones of *P. falciparum*, respectively.

Miliusacunines A (**9**) and B (**10**) were identified from an acetone extract of the leaves and twigs of *Miliusa cuneatas* [33]. Compound **9** demonstrated inhibitory activity against the TM4 malarial strain (IC_50_ 19.3 μM), and compound **10** displayed activity against the K1 malarial strain (IC_50_ 10.8 μM). Both isolates showed no toxicity to the Vero cells at the elevated concentrations.

#### 2.1.2. Araceae Family

**Figure 4:**
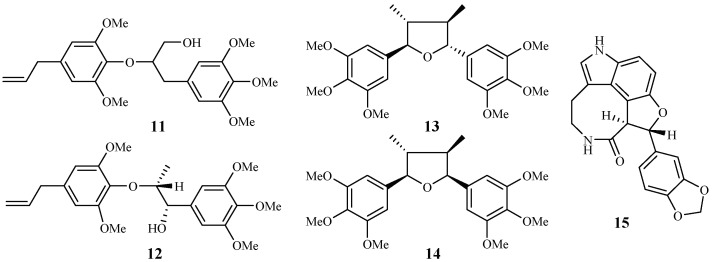
Compounds from an Araceae plant.

Zhang et al. [22,23] performed extensive research on *Rhaphidophora decursiva*, a vine growing in Vietnam. The MeOH extract of the plant leaves and stems showed antimalarial activity against both D6 and W2 clones with no apparent cytotoxicity at a concentration of 20 μg/mL. Seven compounds were identified from the stems and leaves of the plant through a bioassay-guided separation (Figure 4). Polysyphorin (**1**) and rhaphidecurperoxin (**2**) were among the most active compounds, which demonstrated antimalarial activity with IC_50_ values of 1.4–1.8 μM against the D6 and W2 strains and cytotoxicity with ED_50_ values of 8.3–13.1 μM against KB cells (Figure 2). Rhaphidecursinols A (**11**) and B (**12**), grandisin (**13**), epigrandisin (**14**) and decursivine (**15**) also showed activities against *P. falciparum* (D6 and W2) with IC_50_ values of 3.4–12.9 μM and cytotoxicity of ED_50_ values of 23.9–37.0 μM against KB cells with an exception of compound **14**, which showed no antimalarial activity against D6 strain at 23 μM.

According to the further investigation of Zhang et al. [24], a potent but toxic trichothecene compound, roridin E (**3**), was identified from the same plant extract (Figure 2). The investigators determined that the compound was able to inhibit parasite growth with IC_50_ values in the sub-nano molar range. However, roridin E was also very cytotoxic against KB cells. Interestingly, these researchers reported another trichothecene compound (**4**) from a plant in a different family, and the compound showed equally potent antimalarial activities as that of roridin E, but with much less cytotoxicity (see Section 2.8.3).

#### 2.1.3. Asclepiadaceae Family

**Figure 5:**
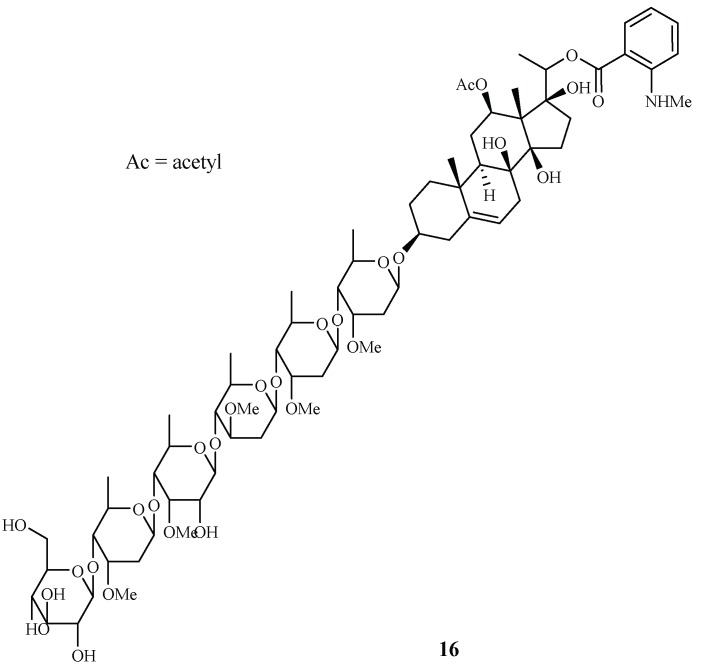
Compound from an Asclepiadaceae plant.

Libman et al. reported the antimalarial bioassay-directed separation of *Gongronema napalense*, leading to the identification of a new steroidal glycoside, gongroneside A (**16**) (Figure 5) [25]. The compound showed inhibitory activity against the D6 and W2 clones with IC_50_ values of 1.6 and 1.4 μM, respectively. Gongroneside A showed no cytotoxicity against KB cells at a concentration of 13.7 μM.

#### 2.1.4. Asteraceae Family

**Figure 6:**
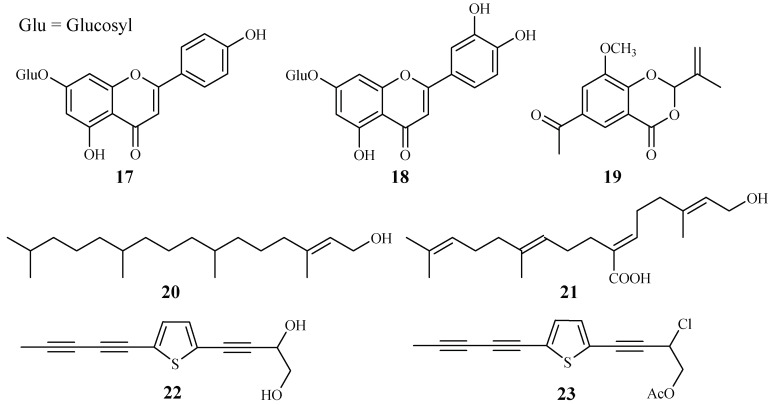
Compounds from Asteraceae plants.

Apigenin 7-*O*-glucoside (**17**) and luteolin 7-*O*-glucoside (**18**), two flavonoid glycosides obtained from the aerial parts of *Achillea millefolium*, showed antiplasmodial activities against D10 and W2 strains with IC_50_ values in the range of 15.3–62.5 μM [34] (Figure 6).

2-Isopropenyl-6-acetyl-8-methoxy-1,3-benzodioxin-4-one (**19**), isolated from the whole plants of the Korean folk medicine *Carpesium divaricatum*, was reported to show antimalarial activity [35,93] (Figure 6). The compound exhibited activity against D10 with an IC_50_ value of 2.3 µM.

*Microglossa pyrifolia*, a medicinal plant used against malaria in Ghana, was tested against both PoW and Dd2 strains of *P. falciparum* by Köhler et al. [36]. Two diterpenes, *E*-phytol (**20**) (IC_50_: 8.5 μM (PoW); 11.5 μM (Dd2)), and 6*E*-geranylgeraniol-19-oic acid (**21**) (IC_50_: 12.9 μM (PoW); 15.6 μM (Dd2)) were shown to be the most active compounds in their test system (Figure 6).

A *Plasmodium berghei*-infected mouse model was used to evaluate the antimalarial activity of the 80% methanol extract of the roots of the traditionally used antimalarial plant *Echinops hoehnelii*. The methanol extract could suppress the parasite growth by 68.5% at a dose of 200mg/kg. No acute oral toxicity was observed in the animal study, indicating the safety use of the plant extract. Further phytochemical separation of the plant led to the isolation of two acetylenicthiophenes, 5-(penta-1,3-diynyl)-2-(3,4-dihydroxybut-1-ynyl)-thiophene (**22**) and 5-(penta-1,3-diynyl)-2-(3-chloro-4-acetoxy-but-1-yn)-thiophene (**23**), which displayed significant growth suppression of the *Plasmodium* parasite by 50.2% and 32.7% at 100 mg/kg, respectively [37] (Figure 6).

### 2.2. Buxaceae Family

**Figure 7:**
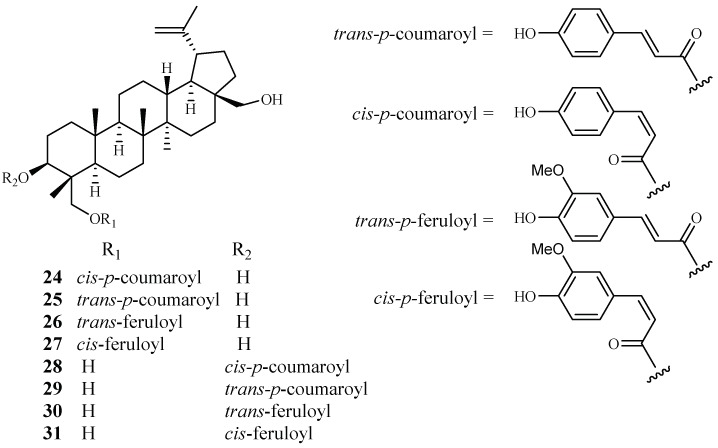
Compounds from a Buxaceae plant.

Cai et al. identified several new antimalarial compounds from *Buxus sempervirens* [38], the native and introduced plant species in the United States. The traditionally used plants have received scant attention as potential source materials for drug discovery research as compared to the botanical materials from tropical and semitropical areas of the world. The eight lupane triterpenes (**24**–**31**), isolated from the *Buxus* plant (Figure 7), were evaluated for their activity against multi-drug-resistant malaria parasites (HB3, IC_50_ 0.5–3.0 μM) and counterscreened against HeLa cells (IC_50_ 7 μM for **24**; >20 μM for **25**–**31**). Strikingly, 23-*O*-(*trans*)-feruloyl-23-hydroxybetulin (**26**) displayed antimalarial activity at a concentration that was 75-fold more selective to the drug-resistant parasite strain than to HeLa cells.

### 2.3. Cecropiaceae–Cucurbitaceae Families

#### 2.3.1. Cecropiaceae Family

**Figure 8:**
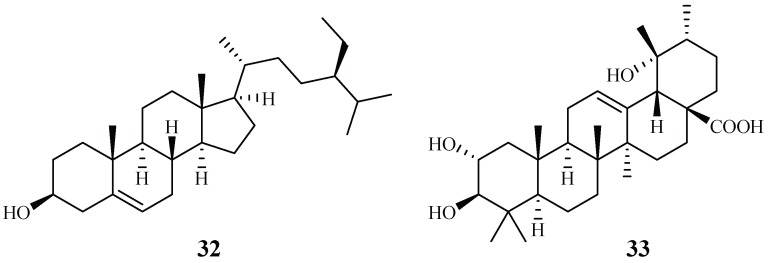
Compounds from a Cecropiaceae plant.

*Cecropia pachystachya* is a medicinal plant, which has been used in Brazil. The ethanol extracts of the different parts of the plants were evaluated for their activity against *P. falciparum* in vitro and *P. berghei* in vivo [39]. The parasitemia of malaria-infected mice was reduced by 35–66% with treatment of the ethanol extracts of the wood, root, and leaf materials in comparison with the non-treated control group. The plant root extracts were further analyzed and fractionated to provide subfractions, which were also active in an in vivo study. Two compounds, β-sitosterol (**32**) and tormentic acid (**33**), were identified from the subfractions (Figure 8). Both compounds showed plasmodial inhibitory activity. However, only tormentic acid (**33**) demonstrated inhibitory activity against *P. falciparum* chloroquine-resistant parasites (W2) (IC_50_ 19.0–25.2 µM).

#### 2.3.2. Chloranthaceae Family

**Figure 9:**
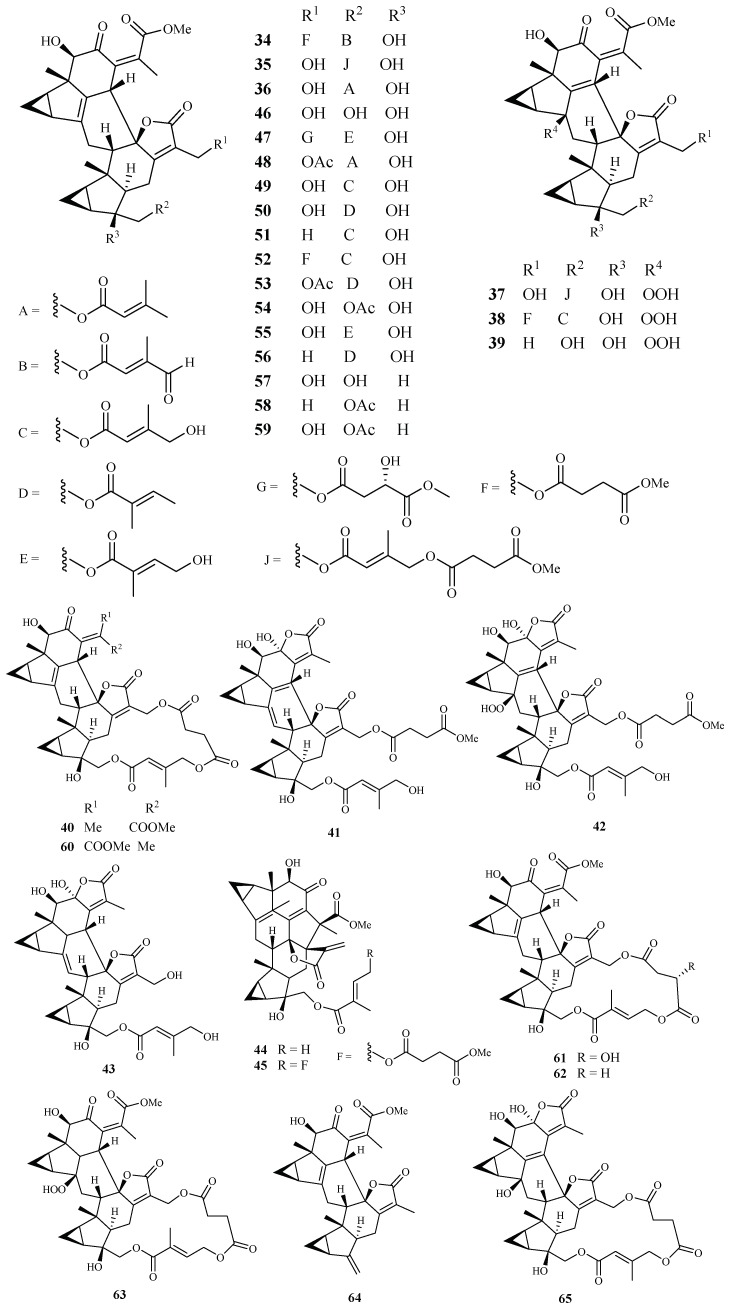
Compounds from Chloranthaceae plants.

Yue et al. [40] recently reported the isolation of 32 antimalarial lindenane-type sesquiterpenoids (**34**–**65**) from several plants in Chloranthaceae family with IC_50_ values lower than 11.4 µM against *P. falciparum* strain Dd2. The 12 new sesquiterpenoid dimers fortunilides A−L (**34**–**45**), along with 7 known isolates (**46**–**51** and **53**) were isolated from *C. fortune*. Compounds **52**, **54**, **58**, **59** and **60**–**64** were obtained from *C. serratus* and *C. spicatus*, and compounds **55**–**57** were separated from *Sarcandra glabra*. Compound **65** was originated from *C. multisachys*. Among these isolates, fortunilide A (**34**), sarglabolide J (**47**) and chlorajaponilide C (**52**) exhibited low nanomolar activities with IC_50_ values of 5.2, 7.2 and 1.1 nM, respectively, and their selectivity index values toward mammalian cells were greater than 500 (Figure 9).

#### 2.3.3. Chrysobalanaceae Family

**Figure 10:**
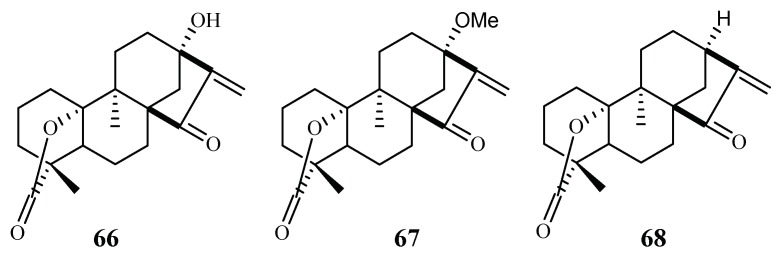
Compounds from a Chrysobalanaceae plant.

From the Petroleum ether/CH_2_Cl_2_ extracts of the stems of *Parinari capensis*, three kaurene diterpene lactones, 10, 13-dihydroxy-9-methyl-15-oxo-20-norkaur-16-en-18-oic acid γ-lactone (**66**), 10-hydroxy-13-methoxy-9-methyl-15-oxo-20-norkaur-16-en-18-oic acid γ-lactone (**67**) and 10-hydroxy-9-methyl-15-oxo-20-norkaur-16-en-18-oic acid γ-lactone (**68**) were isolated (Figure 10) [41]. They possess antimalarial activity against FCR-3 with IC_50_ values of 1.7, 1.9 and 5.0 µM, respectively.

The three compounds (**66**–**68**) also displayed cytotoxicity against Graham cells with ED_50_ values in the range of 3.2–9.2 µM, which preclude them from further biological investigation. They could, however, be used effectively as lead compounds for drug optimization through synthesis.

#### 2.3.4. Clusiaceae Family

**Figure 11:**
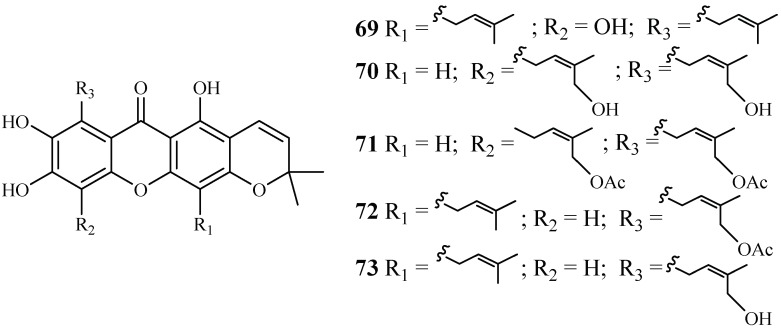
Compounds from a Clusiaceae plant.

Phytochemical separation of the concentrated acetone extract of the dried leaves and branches of *Garcinia mckeaniana* has led to the identification of three new xanthones, mckeanianones A-C (**69**–**71**), and two known ones, bannaxanthones I (**73**) and E (**73**) (Figure 11). These compounds all contain two isoprene units. They were evaluated for their activity against the TM4 and K1 strains of *P. falciparum* with IC_50_ values in the range of of 6.0–8.5 and 3.6–7.3 µM, respectively, and compounds **70**, **71** and **73** showed cytotoxicity against Vero cells with the IC_50_ values in the range of 12.6–29.5 µM [42].

#### 2.3.5. Connaraceae Family

**Figure 12:**
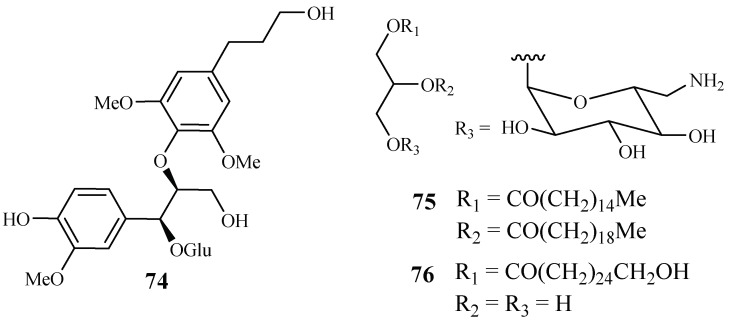
Compounds from a Connaraceae plant.

From the work of He et al. [26], bioassay-guided separation of the chloroform extract of the stems of *Rourea minor* (Gaertn.) Aubl. led to the identification of three active compounds including two new neolignan glycosides, rourinoside (**74**) and rouremin (**75**), and the known 1-(26-hydroxyhexacosanoyl)-glycerol (**76**) (Figure 12). The three compounds showed weak to moderate in vitro activities against the D6 and W2 clones of *P. falciparum*. Compound **74** demonstrated IC_50_ values at 3.7 (D6) and 2.1 (W2) µM; **75** at IC_50_ values of 5.1 (D6) and 4.5 (W2) µM, and **76** at IC_50_ values of 9.5 (D6) and 12.7 (W2) µM. These compounds exhibited no cytotoxicity against KB cells at 20 µg/mL.

#### 2.3.6. Cornaceae Family

**Figure 13:**
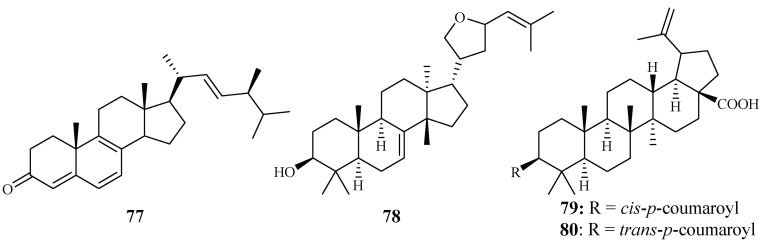
Compounds from a Cornaceae plant.

In vitro IC_50_ values against the *P. falciparum* D10 strain were determined for ergosta-4,6,8,22-tetraene-3-one (**77**) (61.0 µM), 3-epideoxyflindissol (**78**) (128.0 µM), 3β-*O*-*cis*-coumaroyl betulinic acid (**79**) (10.4 µM) and 3β-*O*-*trans*-coumaroyl betulinic acid (**80**) (15.3 µM) (Figure 13), which were separated from the leaves of *Cornus florida* L. by Graziose et al. for the first time [43].

#### 2.3.7. Cucurbitaceae Family

**Figure 14:**
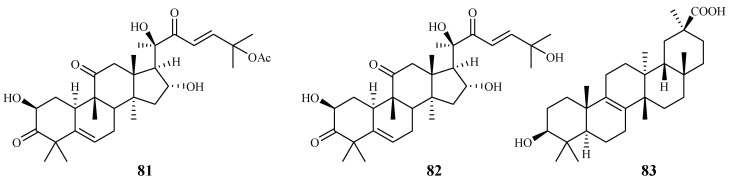
Compounds from a Cucurbitaceae plant.

*Cogniauxia podolaena* Baill. is a folk medicine that has been traditionally used to treat malaria in Congo Brazzaville. Banzouzi et al. [44] identified cucurbitacins B (**81**) and D (**82**), and 20-epibryonolic acid (**83**), the three triterpenes from the stems of this plant (Figure 14). These compounds exhibited inhibitory activity against FcM29 strain with IC_50_ values of 2.9, 7.8 and 4.4 µM, respectively. Both cucurbitacins B and D showed a high cytotoxicity with approximately 95% inhibition against KB cells at 1 μg/mL, while 20-epibryonolic acid displayed a better selectivity index (20% inhibition of KB cells at 1 μg/mL).

### 2.4. Ebenaceae–Euphorbiaceae Families

#### 2.4.1. Ebenaceae Family

**Figure 15:**
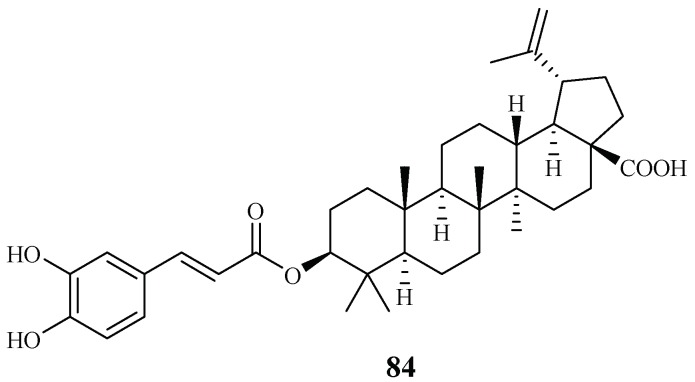
Compounds from an Ebenaceae plant.

Ma et al. [27] investigated the plant *Diospyros quaesita* Thw., known as “Muang Kout” in Laos. Of the isolates from the up parts of this plant, betulinic acid 3-caffeate (**84**) demonstrated antiplasmodial activity against the D6 and W2 clones with IC_50_ values of 1.40 and 0.98 μM, respectively (Figure 15). The compound was cytotoxic to KB cells with an ED_50_ value of 4.0 μM.

#### 2.4.2. Euphorbiaceae Family

**Figure 16:**
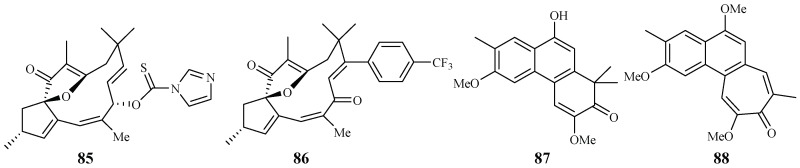
Compounds from Euphorbiaceae plants.

Through the screening of a natural product-based synthetic compound library, Hadi et al. [45] discovered that jatrophones (the natural products from *Jatropha isabelli*) possess significant antiplasmodial activity. The jatrophone diterpene derivatives **85** and **86** displayed antiplasmodial activities against strains 3D7 and K1 of *P. falciparum* with IC_50_ values of 5.7/5.9 and 6.1/5.9 μM, respectively (Figure 16). The two compounds showed low cytotoxicities against the human HepG2, RAJI, BJ and HEK293 cells with EC_50_ values at around 26 μM.

Seephonkai et al. [46] studied the Thai traditional medicinal plant *Strophioblachia fimbricalyx*, and isolated 9-*O*-demethyltrigonostemone (**87**) and a new phenanthropolone, 3,6,9-trimethoxyphenanthropolone (**88**), which exhibited antimalarial activity against the multiresistant K1 strain of *P. falciparum* with IC_50_ values of 8.7 and 9.9 μM, respectively (Figure 16).

### 2.5. Fabaceae–Fagaceae Families

#### 2.5.1. Fabaceae Family

**Figure 17:**
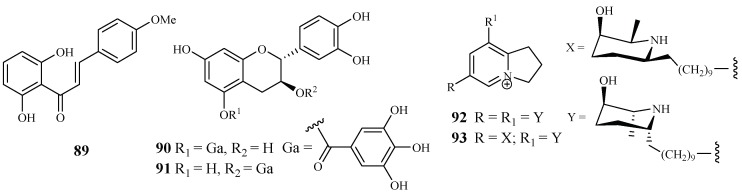
Compounds from Fabaceae plants.

According to Nigerian ethnobotany, the plant *Cajanus cajan* L. (Fabaceae) can be used for treatment of malaria. From the methanol extract of the leaves of this plant, 2′,6′-dihydroxy-4-methoxy chalcone (**89**), a cajachalcone, was isolated through bioassay-guided fractionation, which used the parasite lactate dehydrogenase assay by targeting the K1 strain of *P. falciparum* (Figure 17). The cajachalcone showed an IC_50_ value of 7.4 μM [47].

From the work of Ramanandraibe et al. [48], *Piptadenia pervillei* Vatke was prioritized as an active plant lead identified through a screening program, which was dedicated to discovering antimalarial compounds from the plants in Madagascar. Separation of the EtOAc extract of the leaves of this plant led to the identification of the bioactive compounds (+)-catechin 5-gallate (**90**) and (+)-catechin 3-gallate (**91**). The two compounds showed antimalarial activity against FcB1 strain with IC_50_ values of 1.2 and 1.0 μM, respectively (Figure 17), and no significant cytotoxicity was observed at 75 μM for the two compounds against the human embryonic lung cells MRC-5.

According to the work of Samoylenko et al. [49], prosopilosidine (**92**) and isoprosopilosidine (**93**), isolated from the leaves of *Prosopis glandulosa* var. *glandulosa*, showed potent antimalarial activity against the D6 and W2 strains of *P. falciparum* with high selectivity index (SI) values (Figure 17). Compound **92** exhibited IC_50_ values of 0.1 (D6) and 0.3 (W2) μM, while **93** demonstrated IC_50_ values of 0.1 (D6) and 0.3 (W2) μM. Compounds **92** and **93** showed much lower cytotoxicity to KB cells with ED_50_ values of 20.2 and 18.8 μM, respectively.

#### 2.5.2. Fagaceae Family

**Figure 18:**
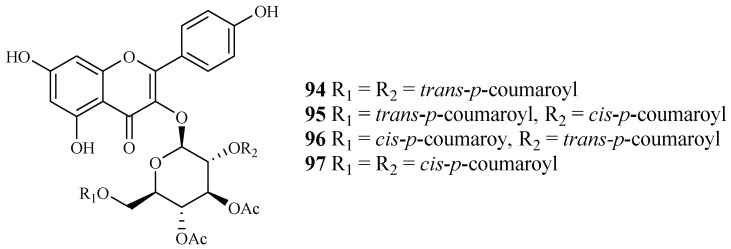
Compounds from a Fagaceae plant.

Subsequent bioassay-guided fractionation work by Cai et al. [38] yielded four kaempferol 3-*O*-glucosides (**94**–**97**) from *Quercus laceyi* (Figure 18). The IC_50_ values for these compounds against multi-drug-resistant malaria parasites HB3 are 0.6–2.1 μM, and the IC_50_ value against HeLa cells was <3 μM.

### 2.6. Hypericaceae Family

*Vismia orientalis*, a traditional medicine used in Tanzania, was studied by Mbwambo et al. [50]. Vismione D (**98**), isolated from the stem barks of this plant, exhibited activity against the K1 strain with an IC_50_ value of 2.4 μM (Figure 18). However, the compound also showed cytotoxicity against human L6 cells with an IC_50_ value of 10.0 μM.

**Figure 19:**
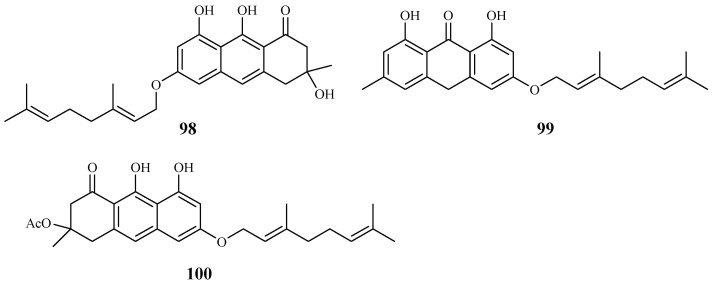
Compounds from Hypericaceae plants.

Pure isolates from the hexane extract of the stem barks of the African plant *Psorospermum glaberrimum* were evaluated for their antimalarial activity against the W2 clone of *P. falciparum* by Ndjakou Lenta et al. [51]. The isolates 3-geranyloxyemodin anthrone (**99**) and acetylvismione D (**100**) displayed inhibition activity against the W2 strain with IC_50_ values of 1.7 and 0.1 µM, respectively (Figure 19).

### 2.7. Lamiaceae–Lythraceae Families

#### 2.7.1. Lamiaceae Family

**Figure 20:**
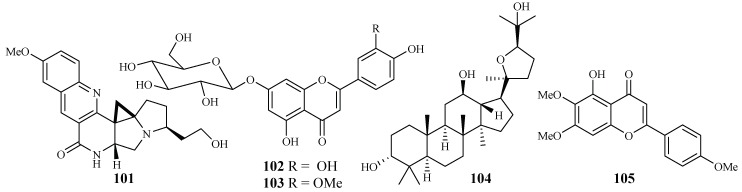
Compounds from Lamiaceae plants.

An EtOH extract of the dried root barks of *Ocimum sanctum* exhibited considerable in vitro antimalarial activity. Bioactivity-directed separation of the EtOH extract resulted in the isolation of a new antimalarial natural compound (**101**) (Figure 20). The compound showed comparable activity to the positive controls, chloroquine and amodiaquine, against the *P. falciparum* 3D7 strains with an IC_50_ value of 0.1 μM [52].

From the study of Kirmizibekmez et al. [53], luteolin 7-*O*-β-d-glucopyranoside (**102**) and chrysoeriol 7-*O*-β-d-glucopyranoside (**103**), two flavonoid glycosides isolated as the major antimalarial constituents from *Phlomis brunneogaleata* through an activity-directed separation (Figure 20), showed activity with IC_50_ values of 5.4 and 12.7 μM against the K1 clones, respectively.

The extracts of 17 *Salvia* species, which are used as folk medicines in South Africa, were subjected to biological testing by Kamatou et al. [54]. The potential activity of the *Salvia* plant extracts against the FCR strain of *P. falciparum* and their cytotoxic effects against MCF-7 cells were investigated. These extracts showed antiplasmodial activity with IC_50_ values in the range of 3.9–26.0 µg/mL. The extracts from *S. radula* demonstrated the most potent activities. Two compounds, betulafolientriol oxide (**104**) and salvigenin (**105**), were subsequently isolated (Figure 20), and they showed antimalarial activity with IC_50_ values of 10.4 and 75.0 μM, respectively.

#### 2.7.2. Loganiaceae Family

**Figure 21:**
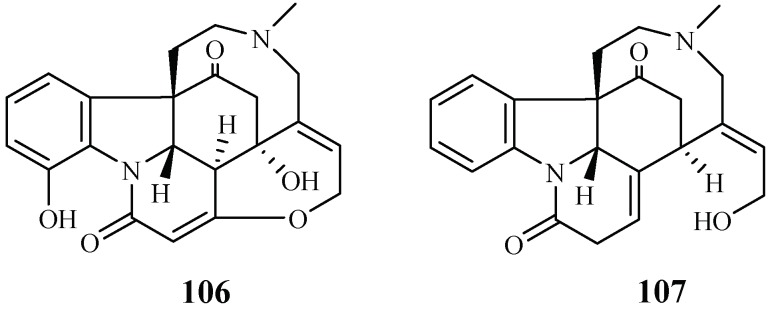
Compounds from a Loganiaceae plant.

A phytochemical study was carried out for the stem barks of *Strychnos icaja* for the first time by Tchinda et al. [55], which led to the isolation of the monomers 15-hydroxyvomicine (**106**) and *N*-methyl-sec-iso-pseudostrychnine (**107**). The isolates were evaluated against the *P. falciparum* 3D7 strain with IC_50_ values of 101.0 and 110.6 μM, respectively (Figure 21).

#### 2.7.3. Lythraceae Family

**Figure 22:**
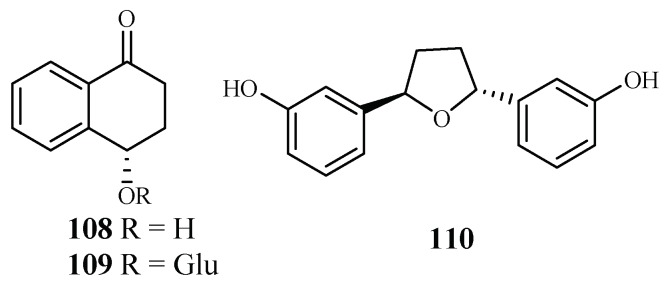
Compounds from Lythraceae plants.

The plants in the genus of *Ammannia* are frequently used in China and India as folk medicines for treatment of various diseases. Upadhyay et al. [56] investigated the compounds in four species of this genus (*Ammannia*: *A. multiflora*, *A. verticillata*, *A. Baccifera* and *A. coccinea*) for their antimalarial activities. Among the isolated compounds, 4-hydroxy-*α*-tetralone (**108**) and tetralone-4-*O*-β-d-glucopyranoside (**109**) from *A. multiflora*, and ammaniol (**110**) from *A. baccifera* displayed antimalarial activities against the *P. falciparum* NF-54 strain with IC_50_ values of 194.0, 124.0 and 88.3 μM, respectively (Figure 22).

### 2.8. Malvaceae–Myristicaceae Families

#### 2.8.1. Malvaceae Family

**Figure 23:**
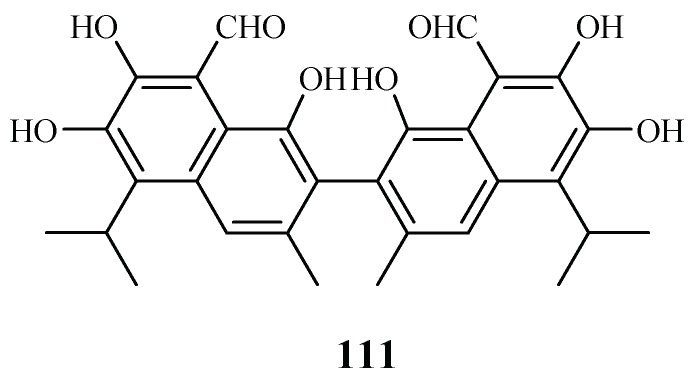
Compounds from a Malvaceae plant.

LC-PDA-MS-SPE-NMR technique was used by Sprogøe et al. in combination with CD to detect (*R*)-(−)-gossypol [(*R*)-1] (**111**) in the twigs of *Thespesia danis* (Figure 23) [57]. (*R*)-1 demonstrated antimalarial activity with an IC_50_ value of 4.5 µM. However, its enantiomer was inactive up to a concentration of 20 µM.

#### 2.8.2. Monimiaceae Family

**Figure 24:**
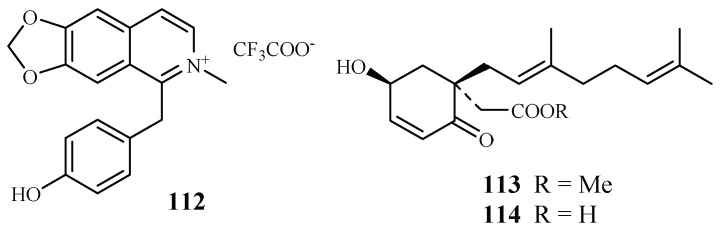
Compounds from Monimiaceae plants.

The compound 1-(4-hydroxybenzyl)-6,7-methylenedioxy-2-methylisoquinolinium trifluoroacetate (**112**), a new benzylisoquinoline alkaloid isolated by mass-guided separation of the CH_2_Cl_2_/MeOH extract of *Doryphora sassafras* (Figure 24) [58]. Compound **112** showed antiplasmodial activity against two different strains (3D7 and Dd2) of *P. falciparum* with IC_50_ values of 3.0 and 4.4 µM, respectively. The compound did not exhibit inhibitory activity against the human embryonic kidney cell line (HEK293) at a concentration of 120 µM.

A phytochemical study of the leaves of *Glossocalyx brevipes* Benth. led to isolation of two new homogentisic acid derivatives of methyl 2-(1′β-geranyl-5′β-hydroxy-2′-oxocyclohex-3′-enyl) acetate (**113**) and 2-(1′β-geranyl-5′β-hydroxy-2′-oxocyclohex-3′-enyl) acetic acid (**114**), which displayed antiplasmodial activity against D6/W2 clones with IC_50_ values of 2.2/6.6 and 4.8/8.3 μM, respectively (Figure 24) [59].

#### 2.8.3. Moraceae Family

According to the investigation of Zhang et al. [24], an antimalarial trichothecene compound, verrucarin L acetate (**4**), was identified from *Ficus fistulosa* (Figure 2). The antimalarial potency of **4** was equivalent to that of roridin E (**3**) isolated from *Rhaphidophora decursiva*, a plant from a different family. However, **4** was observed to be much less cytotoxic to KB cells (ED_50_ 0.2 μM) than **3**.

**Figure 25:**
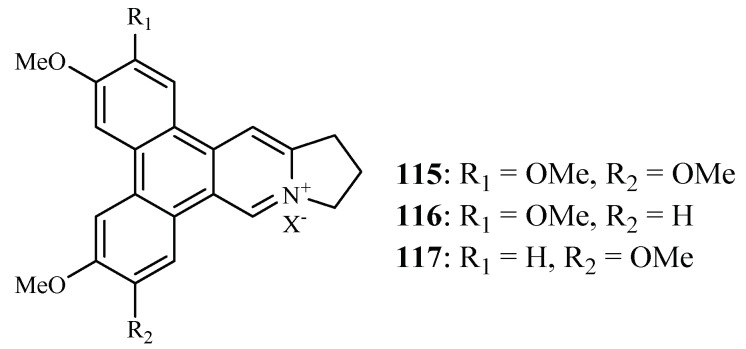
Compounds from a Moraceae plant.

Bioassay-directed separation of the MeOH extract of the twigs of *Ficus septica* afforded three known phenanthroindolizine alkaloids, dehydrotylophorine (**115**), dehydroantofine (**116**) and tylophoridicine D (**117**) by Kubo et al. (Figure 25) [60]. They showed antiplasmodial activity against the *P. falciparum* 3D7 strain with IC_50_ values in the range of 0.03–0.4 µM. Compound **115** also displayed cytotoxicity against the mouse fibroblast cells L929 with an IC_50_ value of 8.2 µM, while the other two compounds showed no toxicity at a concentration of 50 µM.

#### 2.8.4. Myristicaceae Family

**Figure 26:**
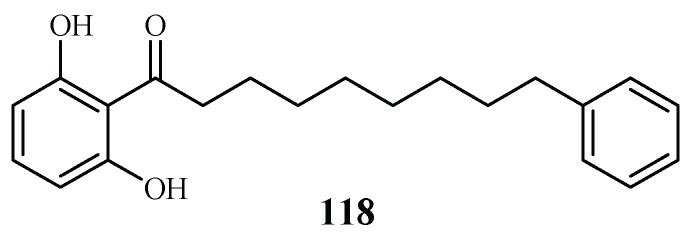
Compound from a Myristicaceae plant.

Phytochemical investigation of the fruits of *Knema glauca* by Rangkaew et al. [61] led to the isolation of malabaricone A (**118**) as an active compound against the *P. falciparum* K1 strain with an IC_50_ value of 8.5 μM (Figure 26). The compound was cytotoxic towards KB cell with an ED_50_ value of >61 μM.

### 2.9. Piperaceae–Platanaceae Families

#### 2.9.1. Piperaceae Family

**Figure 27:**
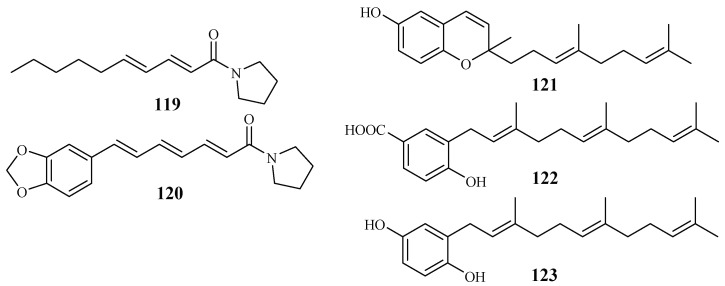
Compounds from Piperaceae plants.

The *Piperaceae* family consists of many plants that are used by the native populations in Thailand as traditional medicines for the treatment of various diseases. Sarmentine (**119**) and 1-piperettyl pyrrolidine (**120**) were isolated from the fruits of *Piper sarmentosum* by Rukachaisirikul et al. [62], and they exhibited antiplasmodial activity against the K1 strain with IC_50_ values of 85.5 and 21.9 μM, respectively (Figure 27).

From the whole plant of *Piper tricuspe*, dictyochromenol (**121**), 3-farnesyl-*p*-hydroxy benzoic acid (**122**) and 2′*E*,6′*E*-2-farnesyl hydroquinone (**123**) were isolated by Saez Vega et al. [63] (Figure 27). The compounds are active against several strains of *P. falciparum* with IC_50_ values ranging from 1.4 to 29.8 µM. Cytotoxic effects were also observed for the compounds with EC_50_ values in the range of 1.1–41.0 µM. The results suggest that the antimalarial activity of the compounds was most probably the direct result of their cytotoxicity.

#### 2.9.2. Platanaceae Family

**Figure 28:**
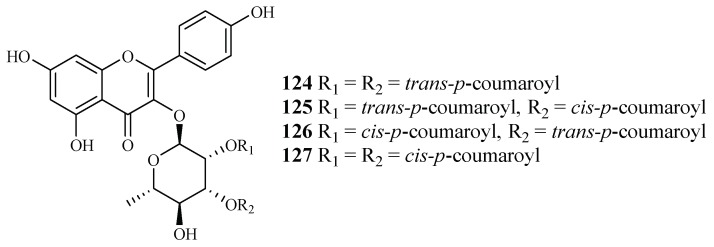
Compounds from a Platanaceae plant.

Bioactivity-guided fractionation of *Platanus occidentalis* by Cai et al. [38] yielded four kaempferol 3-*O*-rhamnosides (**124**–**127**) (Figure 28). The IC_50_ values for these compounds against multi-drug-resistant malaria parasites HB3 ranged from 0.5 to 1.8 µM. The IC_50_ values against HeLa cells were in the range of 9.3–20.0 µM.

### 2.10. Rubiaceae-Rutaceae Families

#### 2.10.1. Rubiaceae Family

**Figure 29:**
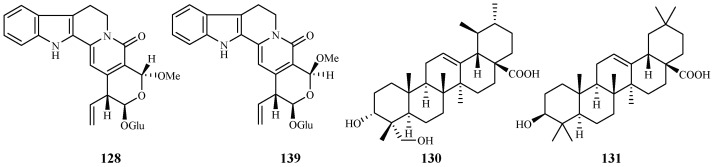
Compounds from a Rubiaceae plant.

Naucleaorine (**128**), epimethoxynaucleaorine (**129**), 3α,23-dihydroxyurs-12-en-28-oic acid (**130**) and oleanolic acid (**131**) were identified from the stems of *Nauclea orientalis* by He et al. [28] (Figure 29). The compounds showed antiplasmodial activities against the *P. falciparum* D6(*)/W2(**) strains with the IC_50_ values shown as below: compound **128** (IC_50_ 6.9*/6.0** µM); **129** (IC_50_ 12.4*/13.2** µM); **130** (IC_50_ 9.7*/12.7** µM) and **131** (IC_50_ 4.6*/5.1** µM). Compounds **128**–**131** displayed cytotoxicity against KB cells with ED_50_ values of 38.0, >37.9, >42.2 and 46.0 µM, respectively.

#### 2.10.2. Rutaceae Family

Based on an ethnomedicinal survey of the plants in Uganda, *Citropsis articulata* was selected for phytochemical study to investigate its antimalarial constituents [64]. From the ethyl acetate extract of the root barks of this plant, two known alkaloids, 5-hydroxynoracronycine (**132**) and 1,5-dihydroxy-2,3-dimethoxy-10-methyl-9-acridone (**133**), were identified as the best growth inhibitors of *P. falciparum* with IC_50_ values of 2.8 and 10.0 μM, respectively. The compounds were cytotoxic towards Vero cells at EC_50_ values of 28.8 and 101.0, respectively.

**Figure 30:**
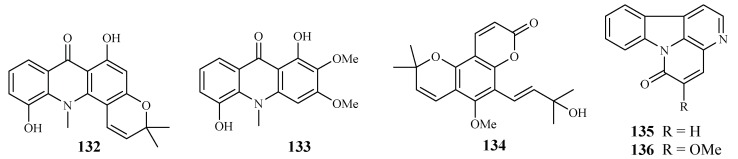
Compounds from Rutaceae plants.

The roots and stem barks of *Zanthoxylum chiloperone* have been used as a folk medicine for the treatment of malaria and for its emmenagogue and antirheumatic properties. The pyranocoumarin *trans*-avicennol (**134**) and two canthinone alkaloids, canthin-6-one (**135**) and 5-methoxycanthin-6-one (**136**), were identified from the stem barks of this plant by Cebrián-Torrejón et al. [65] (Figure 30). These compounds possessed antiplasmodial IC_50_ values against chloroquine/mefloquine resistant and sensitive strains of *P. falciparum* (F32, K1, PFB and FcB1 cells) in the range of 1.4–41.6 μM. Compounds **134** and **135** were cytotoxic towards MCR5 cells with EC_50_ values of 12.8 and 42.7 μM, respectively.

### 2.11. Simaroubaceae Family

**Figure 31:**
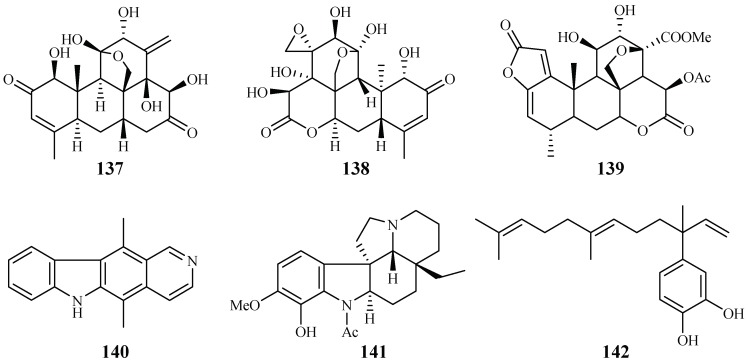
Compounds from Simaroubaceae plants.

Kuo et al. [66] found that among the isolates from the roots of *Eurycoma longifolia*, eurycomanone (**137**) and pasakbumin B (**138**) [67] displayed potent antimalarial activity against the *P. falciparum* W2/D6 strains with IC_50_ values of 0.04/0.06 and 0.05/0.08 μM, respectively (Figure 31). The compounds also exhibited strong cytotoxicity toward human breast cancer (MCF-7) and lung cancer (A549) cells at low concentrations.

De Andrade-Neto et al. [68] studied a number of Simaroubaceous plants, resulting in the isolation of the following compounds: the quassinoid neosergeolide (**139**) from the roots and stems of *Picrolemma spruce* (Figure 31); the indole alkaloids ellipticine (**140**) and aspidocarpine (**141**) from the barks of *Aspidosperma vargasii* and *A. desmanthum* (Apocynaceae), respectively; and 4-nerolidylcatechol (**142**) from the roots of *Pothomorphe peltata* (Piperaceae). Compounds **139**–**141** presented significant inhibitory activity against the multi-drug resistant K1 strain with IC_50_ values of 0.002, 0.07, 0.02 and 0.7 μM, respectively, and these compoundsdisplayed antimalarial potency greater than those of quinine and chloroquine.

### 2.12. Theaceae–Tiliaceae Families

#### 2.12.1. Theaceae Family

**Figure 32:**
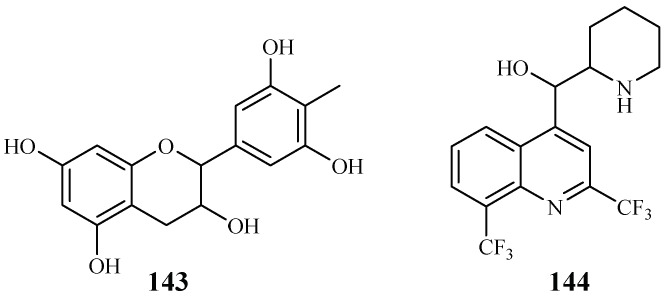
Compound from Theaceae plants.

Gallocatecin (**143**) is a flavonoid contained in the tea leaf extract of *Camellia sinensis* (Figure 32). Based on molecular docking studies, Tegar et al. [69] found that gallocatecin has stronger antimalarial potency than mefloquine (**144**), a synthetic drug with antimalarial activity.

#### 2.12.2. Tiliaceae Family

**Figure 33:**
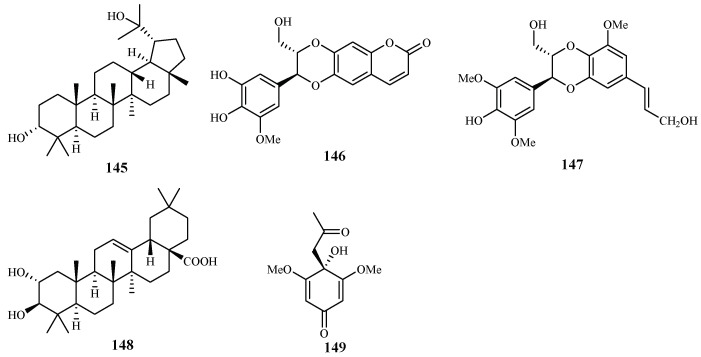
Compounds from a Tiliaceae plant.

According to the study of Ma et al. [29], five isolates from the leaves, stems and twigs of *Grewia bilamellata*, 3*α*,20-lupandiol (**145**), grewin (**146**), nitidanin (**147**), 2*α*,3β-dihydroxyolean-12-en-28-oic acid (**148**) and 2,6-dimethoxy-1-acetonylquinol (**149**), displayed antimalarial activity against the *P. falciparum* D6 and W2 clones with IC_50_ values in the range of 5.5–42.2 µM (Figure 33). These compounds showed no cytotoxicity towards KB carcinoma cell line at a concentration of 50 µM.

### 2.13. Verbenaceae Family

**Figure 34:**
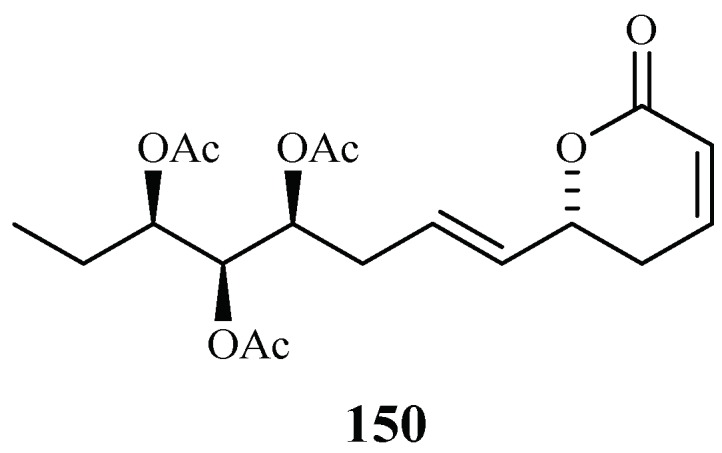
Compound from a Verbenaceae plant.

Chromatographic separation of the ethyl acetate extract of the aerial parts of *Lippia javanica* yielded a new antimalarial *α*-pyrone, lippialactone (**150**) (Figure 34). This compound is active against the D10 strain with an IC_50_ value of 23.8 μM. Compound **119** is also mildly cytotoxic [70].

## 3. Marine Plant-Derived Antimalarial Compounds

Marine organisms offer unique opportunity to discover lead compounds for the treatments of malaria.

**Figure 35:**
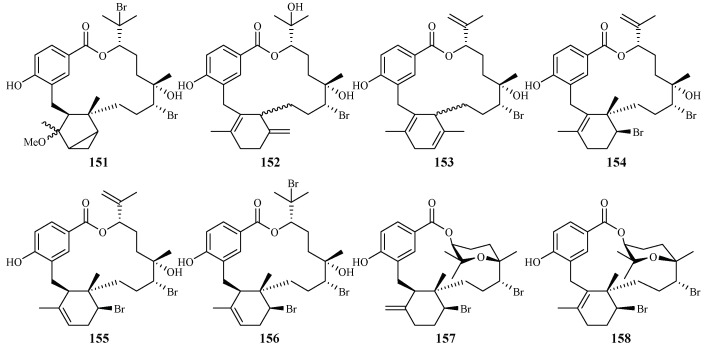
Compounds (**151**–**158**) from the red alga *Callophycus serratus*.

**Figure 36:**
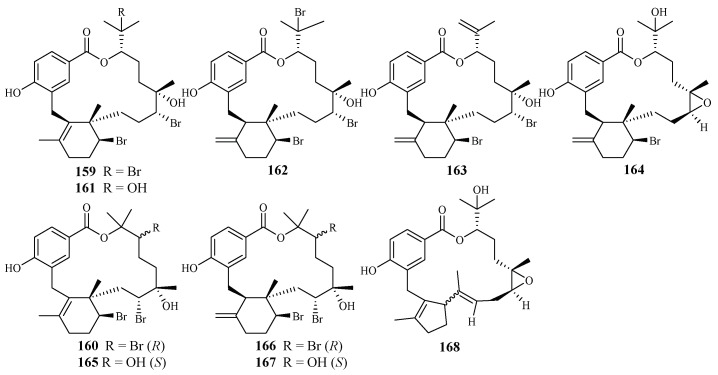
Compounds (**159**–**168**) from the red alga *Callophycus serratus*.

Separation of the extracts of Fijian red alga *Callophycus serratus* by Lane et al. led to the isolation of bromophycolides J-Q (**151**–**158**) [94] (Figure 35), the macrolide diterpene-benzoate derivatives represented as two novel carbon skeletons. These diterpenes, together with the previously reported ten bromophycolides, bromophycolides A-I (**159**–**167**) and debromophycolide A (**168**) from this alga (Figure 36) [95], were evaluated for their antimalarial activity against *P. falciparum*. The IC_50_ values of bromophycolides A, D, E, H and M (**159**, **162**, **163**, **164** and **154**) were observed to be 0.9, 0.3, 0.8, 0.9 and 0.5 µM, respectively. Some of these compounds also exhibited strong cytotoxicity toward DU4475, a human breast cancer cell line. The ED_50_ values of bromophycolides N and Q (**155** and **158**) were 1.5 and 2.0 µM, respectively.

**Figure 37:**
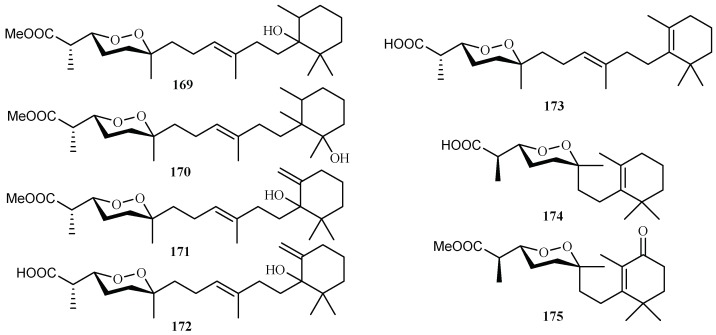
Compounds from the sponge *Diacarnus megaspinorhabdosa*.

From the sponge *Diacarnus megaspinorhabdosa* collected in Xisha Islands, four new norterpene cyclic peroxides, diacarnuperoxides M (**169**) and N (**170**), (+)-2, 3, 6-epihurghaperoxide (**171**) and (+)-2,3,6-epihurghaperoxide acid (**172**), together with the known norterpene cyclic peroxides, (−)-muqubilin A (**173**), nuapapuin A (**174**) and diacarperoxide A (**175**) were isolated by Yang et al. [96] (Figure 37). They exhibited inhibitory activity against W2 clones of the malaria parasite *P. falciparum* with IC_50_ values of 4.2, 3.0, 1.6, 4.9, 5.6, 5.5 and 1.6 µM, respectively.

## 4. Ethnologic Antimalarial Compounds

At present, more than 80% of the world’s population relies on ethnopharmacologic healing modalities and plants for their primary health care and wellness [97]. In Africa and many other developing countries, ethnomedicines are often regarded as their primary choice to treat diseases as they are obtained most affordable and accessible from locally available plants or other natural sources [78]. Plants are the major resource for the treatment of malaria infections in sub-Saharan Africa, where health care facilities are limited [98]. Ethnomedicinal plants have played a pivotal role in the treatment of malarial for centuries [71,99].

Early writing of over 6000 years ago in Egypt and China, and those of the Vedic civilisation dated 1600 B.C. in India, indicate that malaria has afflicted humans since antiquity, and there is ample evidence that antimalarial traditional medicaments have been used in virtually all cultures as the mainstay for the treatment of this disease. In the 5th century B.C., Hippocrates rejected superstition as a cause for the fevers that afflicted ancient Greeks. He instead recognized the seasonality of fevers and described the early clinical manifestations and complication of malaria [71].

The widely used antimalarial drug, artemisinin, was isolated from the traditional Chinese herb *Artemisia annua* L. (Qinghao) [11], which has been used in China as an ethnomedicine for close to 2000 years. The treatment of malaria with Qinghao was first recorded in “Zhouhou Bei Ji Fang”, the handbook of prescriptions for emergencies in 243 A.D. [71,77].

The use of ethnomedicine such as herbs for the treatment of malaria varies by region, environment and population subgroups. It may be more preferred in some areas than in others. In order to explore the ethnologic basis of these antimalarial plants, several hundred species from 50 families are presently reviewed and listed in Table 3. These plants were collected from 13 countries and areas, exemplified by Madagascar, Nigeria, South Africa and India. The antimalarial activity and toxicity of these plants are also presented in the table [7,64,72,73,74,75,76,79,80,81,82,83,84,85,86,87,88,89,90,91,92,100].

## 5. Conclusions

It is imperative that the search for new antimalarial agents continues at an unabated pace in order to meet the challenges posed by the development of antimalarial drug resistance. During the last decade, numerous antimalarial compounds have been isolated from plants, and many of these compounds exhibit significant activity against *P. falciparum* in vitro. It is, therefore, evident that plant secondary metabolites continue to play an important role in pre-clinical antimalarial drug discovery.

We present in this comprehensive review, the structures of 175 plant-derived antiplasmodial compounds that have been published during the period of 2001–2017. The relevant plants are organized according to the geographical origins of their corresponding plant families.

Among the 175 plant-derived antiplasmodial compounds, several classes of compounds that showed nanomolar range of activity can be regarded as lead compounds to further explore their antimalarial potential. The trichothecene roridin E (**3**) from *Rhaphidophora decursiva* (Araceae family) showed potent inhibitory effects against the parasite growth with IC_50_ values in the sub-nano molar range (IC_50_: 0.4 nM (D6), 1 nM (W2)) with high cytotoxicity against KB cells (ED_50_: 0.4 nM). However, its closely related structural analog, verrucarin L acetate (**4**), identified from *Ficus fistulosa* (Moraceae family), displayed much lower cytotoxicity to KB cells (ED_50_ 200 nM) while retaining the same level of the antiplasmodial activity as **3**. Identified from the plant (*Ficus septica*) in the same genus as that of **4**, the phenanthroindolizine alkaloids dehydroantofine (**116**) and tylophoridicine D (**117**) demonstrated potent antiplasmodial activity against the *P. falciparum* 3D7 strain with IC_50_ values of 30 and 60 nM, respectively, and the compounds showed no toxicity at a concentration of 50 μM. A recent study found that the lindenane-type sesquiterpenoids fortunilide A (**34**), sarglabolide J (**47**) and chlorajaponilide C (**52**) from the plant in Chloranthaceae family displayed potent antiplasmodial activity against Dd2 strain of with IC_50_ values of 5.2, 7.2 and 1.1 nM, respectively, and these compounds also showed low cytotoxicity to the mammalian cells WI-38 with IC_50_ values of 8.8, 4.0 and 5.4 μM, respectivley. More prominently, fortunilide E (**38**) containing a peroxide group showed antiplasmodial activity of 43 nM with no cytotoxicity at 100 μM.

This review also describes 25 antimalarial compounds that were reported from marine plants during the time period covered. In addition, we included ethnologic information on antimalarial plants from 50 families that are used as folk medicines for the treatment of malaria. Taken together, all the information presented attests to the fact that the phytochemical investigation of terrestrial plants coupled with the biological validation of ethnomedicines constitute proven strategies for the discovery of potential lead compounds for antimalarial drug development.

## Figures and Tables

**Table 1:** Available antimalarial drugs.

Chemical Class	Generic Names	Chemical Class	Generic Names
4-Aminoquinolines	chloroquine	Antibiotics	azythromycin
	amodiaquine		clindamycin
	piperaquine		doxycycline
8-Aminoquinoline	primaquine	Artemisinin-based combination therapy (ACT)	artemether-lumefantrine
	bulaquine		artesunate
Arylamino-alcohols	quinine		artesunate/sulfadoxine/pyrimethamine
	quinidine		artesunate/sulfadoxine-pyrimethamine/primaquine
	mefloquine		artesunate/amodiaquine
	halofantrine		artesunate/mefloquine
	lumefantrine		artesunate/pyronaridine
Biguanides	proguanil		chloroquine/primaquine
	chlorproguanil		dihydroartemisinin/piperaquine
Glycosylamines	pyrimethamine	Antibiotics-antimalarial drug combination	doxycyclin/quinine
	proguanil		doxycycline/artesunate
	cycloguanil		doxycyclin/mefloquine
	chlorproguanil		clindamycin/quinine
	chlorcycloguanil		clindamycin/artesunate
Naphthoquinone	atovaquone		clindamycin/mefloquine
Sesquiterpene lactones	artemisinin	Other combination therapy	sulfadoxine/pyrimethamine
	arteether		bulaquine/chloroquine
	artemether		dapsone/chlorproguanil
	artesunate		atovaquone/proguanil
	dihydroartemisinin		
Sulfonamides/Sulfones	sulfadoxine		
	sulfalene		
	dapsone		

**Table 2:** Antiplasmodial activities and toxicities of compounds isolated from terrestrial plants.

Family	Species	Extract Solvent	Compound	Antiplasmodial IC_50_ (μM) ^a^ (*P. falciparum*)	Cytotoxicity, ED_50_ (μM) ^b^ (Cell Line)	References
Annonaceae	*Friesodielsia discolor*	EtOAc	30-formyl-20,40-dihydroxy-60-methoxychalcone (**5**)	9.2 (K1)	21.8 (KB); 13.9 (MCF-7)	[31]
			8-formyl-7-hydroxy-5-methoxyflavanone (**6**)	9.3 (K1)	41.9 (KB); 34.5 (MCF-7)	
			tectochrysin (**7**)	7.8 (K1)	59.1 (KB); 16.8 (MCF-7)	
	*Mitrephora diversifolia*	CH_2_Cl_2_/MeOH	5-hydroxy-6-methoxyonychine (**8**)	9.9 (3D7); 11.4 (Dd2)	120.0 (HEK293)	[32]
	*Miliusa cuneata*	Acetone	miliusacunines A (**9**)	19.3 (TM4)	–	[33]
			miliusacunines B (**10**)	10.8 (K1)	–	
Araceae	*Rhaphidophora decursiva*	MeOH	polysyphorin (**1**)	1.7 (D6); 1.5 (W2)	8.3 (KB)	[22,23]
			rhaphidecurperoxin (**2**)	1.8 (D6); 1.4 (W2)	13.1 (KB)	
			rhaphidecursinol A (**11**)	7.2 (D6); 4.2 (W2)	28.7 (KB)	
			rhaphidecursinol B (**12**)	12.9 (D6); 11.2 (W2)	23.9 (KB)	
			grandisin (**13**)	3.5 (D6); 3.4 (W2)	32.4 (KB)	
			epigrandisin (**14**)	>23 (D6); 7.7 (W2)	37.0 (KB)	
			decursivine (**15**)	11.2 (D6); 12.6 (W2)	–	[22,23]
			Roridin E (**3**)	0.0004 (D6); 0.001 (W2)	0.0004 (KB)	[24]
Asclepiadaceae	*Gongronema napalense*	EtOH	gongroneside A (**16**)	1.6 (D6); 1.4 (W2)	>13.7 (KB)	[25]
Asteraceae	*Achillea millefolium*	MeOH	apigenin 7-*O*-glucoside (**17**)	25.3 (D10); 15.3 (W2)	–	[34]
			luteolin 7-*O*-glucoside (**18**)	61.1 (D10); 62.5 (W2)	–	
	*Carpesium divaricatum*	MeOH	2-isopropenyl-6-acetyl-8-methoxy-1,3-benzodioxin-4-one (**19**)	2.3 (D10)	63.2 (SK-OV-3)	[35]
	*Microglossa pyrifolia*	Petroleum ether-EtOAc (1:1, *v*/*v*)	*E*-phytol (**20**)	8.5 (PoW); 11.5 (Dd2)	–	[36]
			6*E*-geranylgeraniol-19-oic acid (**21**)	12.9 (PoW); 15.6 (Dd2)	–	
	*Echinops hoehnelii*	CH_2_Cl_2_	5-(penta-1,3-diynyl)-2-(3,4-dihydroxybut-1-ynyl)-thiophene (**22**)	50.2% (100 mg/kg)	–	[37]
			5-(penta-1,3-diynyl)-2-(3-chloro-4-acetoxy-but-1-yn)-thiophene (**23**)	32.7% (100 mg/kg)	–	
Buxaceae	*Buxus semperviren*	MeOH	compound (**24**)	0.5–3.0 (HB3)	7.0 (Hela)	[38]
			compound (**25**)	0.5–3.0 (HB3)	>20 (Hela)	
			23-*O*-(*trans*)-feruloyl-23-hydroxybetulin (**26**)	0.5–3.0 (HB3)	>20 (Hela)	
			compound (**27**–**31**)	0.5–3.0 (HB3)	>20 (Hela)	
Cecropiaceae	*Cecropia pachystachya*	EtOH	β-sitosterol (**32**)	>120 (W2)	–	[39]
			tormentic acid (**33**)	19.0–25.2 (W2)	–	
Chloranthaceae	*Chloranthus. fortunei*	EtOH	fortunilide A (**34**)	0.005 (Dd2)	8.8 (WI-38)	[40]
			fortunilide B (**35**)	0.02 (Dd2)	3.1 (WI-38)	
			fortunilide C (**36**)	0.2 (Dd2)	-	
			fortunilide D (**37**)	0.03 (Dd2)	0.5 (WI-38)	
			fortunilide E (**38**)	0.04 (Dd2)	>100 (WI-38)	
			fortunilide F (**39**)	5.3 (Dd2)	-	
			fortunilide G (**40**)	0.05 (Dd2)	1.2 (WI-38)	
			fortunilide H (**41**)	0.2 (Dd2)	-	
			fortunilide I (**42**)	0.09 (Dd2)	-	
			fortunilide J (**43**)	9.9 (Dd2)	-	
			fortunilide K (**44**)	4.7 (Dd2)	-	
			fortunilide L (**45**)	0.1 (Dd2)	15.5 (WI-38)	
			sarglabolide I (**46**)	4.6 (Dd2)	-	
			sarglabolide J (**47**)	0.007 (Dd2)	4.0 (WI-38)	
			shizukaol K (**48**)	0.9 (Dd2)	-	
			shizukaol I (**49**)	0.1 (Dd2)	-	
			shizukaol C (**50**)	0.02 (Dd2)	0.8 (WI-38)	
			schizukaol M (**51**)	0.10 (Dd2)	4.5 (WI-38)	
			chlorahololide D (**53**)	0.01 (Dd2)	0.2 (WI-38)	
	*C. multisachys*	-	chloramultilide B (**65**)	7.1 (Dd2)	-	
	*C. serratus* and *C. spicatus*	-	chlorajaponilide C (**52**)	0.001 (Dd2)	5.4 (WI-38)	
			shizukaol N (**54**)	0.1 (Dd2)	10.0 (WI-38)	
			shizukaol E (**58**)	1.8 (Dd2)	-	
			shizukaol D (**59**)	0.6 (Dd2)	-	
			shizukaol F (**60**)	0.01 (Dd2)	0.2 (WI-38)	
			shizukaol G (**61**)	0.01 (Dd2)	1.7 (WI-38)	
			shizukaol B (**62**)	0.03 (Dd2)	16.7 (WI-38)	
			spicachlorantin D (**63**)	0.5 (Dd2)	-	
			shizukaol A (**64**)	1.5 (Dd2)	-	
	*Sarcandra glabra*	-	sarcandrolide B (**55**)	0.27 (Dd2)	-	
			sarcandrolide A (**56**)	0.3 (Dd2)	-	
			sarcandrolide J (**57**)	11.4 (Dd2)	-	
Chrysobalanceae	*Parinari capensis*	Petroleum ether and CH_2_Cl_2_	10,13-dihydroxy-9-methyl-15-oxo-20-norkaur-16-en-18-oic acid γ-lactone (**66**)	1.7 (FCR-3)	5.5 (Graham)	[41]
			10-hydroxy-13-methoxy-9-methyl-15-oxo-20-norkaur-16-en-18-oic acid γ-lactone (**37**)	1.9 (FCR-3)	3.2 (Graham)	
			10-hydroxy-9-methyl-15-oxo-20-norkaur-16-en-18-oic acid γ-lactone (**68**)	5.0 (FCR-3)	9.6 (Graham)	
Clusiaceae	*Garcinia mckeaniana*	Acetone	mckeanianones A (**69**)	6.2 (TM4)	–	[42]
			mckeanianones B (**70**)	6.7 (TM4)	12.9 (Vero)	
			mckeanianones C (**71**)	6.0 (TM4)	29.5 (Vero)	
			bannaxanthones I (**72**)	8.5 (TM4)	–	
			bannaxanthones E (**73**)	8.3 (TM4)	–	
Connaraceae	*Rourea minor* (Gaertn.) Aubl.	CHCl_3_	rourinoside (**74**)	3.7 (D6); 2.1 (W2)	KB: ED_50_: >35.1	[26]
			rouremin (**75**)	5.1 (D6); 4.5 (W2)	KB: ED_50_: >25.5	
			1-(26-hydroxyhexacosanoyl)-glycerol (**76**)	9.5 (D6); 12.7 (W2)	KB: ED_50_: >41.2	
Cornaceae	*Cornus florida* L.	EtOH	ergosta-4,6,8,22-tetraene-3-one (**77**)	61.0 (D10)	27.0 (L6)	[43]
			3-epideoxyflindissol (**78**)	128.0 (D10)	14.7 (L6)	
			3β-*O*-*cis*-coumaroyl betulinic acid (**79**)	10.4 (D10)	5.6 (L6)	
			3β-*O*-*trans*-coumaroyl betulinic acid (**80**)	15.3 (D10)	9.3 (L6)	
Cucurbitaceae	*Cogniauxia podolaena* Baill.	CH_2_Cl_2_	cucurbitacin B (**81**)	2.9 (FcM29 strain)	94% inhibition of KB at 1.8 μM	[44]
			cucurbitacin D (**82**)	7.8 (FcM29 strain)	95% inhibition of KB at 1.9 μM	
			20-epibryonolic acid (**83**)	4.4 (FcM29 strain)	20% inhibition of KB at 2.2 μM	
Ebenaceae	*Diospyros quaesita* Thw.	CHCl_3_	betulinic acid 3-caffeate (**84**)	1.4 (D6); 1.0 (W2)	4.0 (KB)	[27]
Euphorbiaceae	*Jatropha isabelli*	-	compound **85**	–	–	[45]
			compound **86**	–	–	
	*Strophioblachia fimbricalyx*	MeOH	9-*O*-demethyltrigonostemone (**87**)	8.7 (K1)	2.6 (KB)	[46]
			3,6,9-trimethoxyphenanthropolone (**88**)	9.9 (K1)	12.3 (KB)	
Fabaceae	*Cajanus cajan* L.	-	cajachalcone (**89**)	7.4 (K1)	–	[47]
	*Piptadenia pervillei*	EtOAc	(+)-catechin 5-gallate (**70**)	1.2 (FcB1)	>75 (MRC-5)	[48]
			(+)-catechin 3-gallate (**91**)	1.0 (FcB1)	>75 (MRC-5)	
	*Prosopis glandulosa* var. *glandulosa*	EtOH	prosopilosidine (**92**)	0.1 (D6); 0.3 (W2)	20.2 (KB)	[49]
			isoprosopilosidine (**93**)	0.1 (D6); 0.3 (W2)	18.8 (KB)	
Fagaceae	*Quercus laceyi*	MeOH	kaempferol 3-*O*-glucosides (**94**–**97**)	0.6–2.1 (HB3)	<3.0 (Hela)	[38]
Hypericaceae	*Vismia orientalis*	-	vismione D (**98**)	2.4 (K1)	10.0 (L6 cell)	[50]
	*Psorospermum glaberrimum*	Hexane	3-geranyloxyemodin anthrone (**99**)	1.7 (W2)	–	[51]
			acetylvismione D (**100**)	0.1 (W2)	–	
Lamiaceae	*Ocimum sanctum*	EtOAc	compound **101**	0.1 (3D7)	–	[52]
	*Phlomis brunneogaleata*	MeOH	luteolin 7-*O*-β-d-glucopyranoside (**102**)	5.4 (K1)	>200	[53]
			chrysoeriol 7-*O*-β-d-glucopyranoside (**103**)	12.7 (K1)	>194	
	*Salvia radula*	MeOH:CHCl_3_ = 1:1	betulafolientriol oxide (**104**)	10.4 (FCR-3)	–	[54]
			salvigenin (**105**)	75.0 (FCR-3)	207 (MCF-7)	
Loganiaceae	*Strychnos icaja*	EtOAc-EtOH-NH_4_OH (96:3:1)	15-hydroxyvomicine (**106**)	101.0 (W2)	–	[55]
			*N*-methyl-sec-iso-pseudostrychnine (**107**)	110.6 (W2)	–	
Lythraceae	*Ammannia multiflora*, *A. baccifera*	MeOH	4-hydroxy-α-tetralone (**108**)	194.0 (NF-54)	–	[56]
			tetralone-4-*O*-β-d-glucopyranoside (**109**)	124.0 (NF-54)	–	
			ammaniol (**110**)	88.3 (NF-54)	–	
Malvaceae	*Thespesia danis.*	Acetone–water (7:3)	(*R*)-(−)-gossypol (**111**)	4.5 (3D7)	–	[57]
Monimiaceae	*Doryphora sassafras*	CH_2_Cl_2_/MeOH	1-(4-hydroxybenzyl)-6,7-methylenedioxy-2-methylisoquinolinium trifluoroacetate (**112**)	3.0 (3D7); 4.4 (Dd2)	120.0 (HEK293)	[58]
	*Glossocalyx brevipes* Benth.	CHCl_3_/MeOH (1/1)	methyl 2-(1′β-geranyl-5′β-hydroxy-2′-oxocyclohex-3′-enyl) acetate (**113**)	2.2 (D6); 6.6 (W2)	–	[59]
			2-(1′β-geranyl-5′β-hydroxy-2′-oxocyclohex-3′-enyl) acetic acid (**114**)	4.8 (D6); 8.3 (W2)	–	
Moraceae	*Ficus fistulosa*	-	verrucarin L acetate (**4**)	0.001 (D6); 0.001 (W2)	0.2 (KB)	[24]
	*F. septica*	MeOH	dehydrotylophorine (**115**)	0.4 (3D7)	8.2 (L929)	[60]
			dehydroantofine (**116**)	0.03 (3D7)	>55 (L929)	
			tylophoridicine D (**117**)	0.06 (3D7)	>56 (L929)	
Myristicaceae	*Knema glauca*	EtOAc	malabaricone A (**118**)	8.5 (K1)	>61 (KB); 55.4 (NCI-H187)	[61]
Piperaceae	*Piper sarmentosum*	Hexane-MeOH	sarmentine (**119**)	85.5 (K1)	–	[62]
			1-piperettyl pyrrolidine (**120**)	21.9 (K1)	–	
	*P. tricuspe*	Petroleum ether	dictyochromenol (**121**)	9.6 (FcB1)	7.7 (L-6)	[63]
			3-farnesyl-*p*-hydroxy benzoic acid (**122**)	29.8 (FcB1)	40.9 (L-6)	
			2′*E*,6′*E* 2-farnesyl hydroquinone (**123**)	1.4 (FcB1)	1.1 (L-6)	
Platanaceae	*Platanus occidentalis*	MeOH	kaempferol 3-*O*-rhamnosides (**124**–**127**)	0.5–1.8 (HB3)	9.3–20.0 (Hela)	[38]
Rubiaceae	*Nauclea orientalis*	MeOH	naucleaorine (**128**)	6.9 (D6); 8.0 (W2)	38.0 (KB)	[28]
			epimethoxynaucleaorine (**129**)	12.4 (D6); 13.2 (W2)	>37.9 (KB)	
			3α,23-dihydroxyurs-12-en-28-oic acid (**130**)	9.7 (D6); 12.7 (W2)	>42.2 (KB)	
			oleanolic acid (**131**)	4.6 (D6); 5.1 (W2)	46.0 (KB)	
Rutaceae	*Citropsis articulata*	MeOH	5-hydroxynoracronycine (**132**)	2.8 (FcB1)	28.8 (Vero)	[64]
			1,5-dihydroxy-2,3-dimethoxy-10-methyl-9-acridone (**133**)	10.0 (FcB1)	101 (Vero)	
	*Zanthoxylum chiloperone* var. *angustifolium* Engl.	CH_2_Cl_2_	*trans*-avicennol (**134**)	7.8 (K1); 1.5 (F32); 3.5 (PFB); 6.4 (FcB1)	12.8 (MCR5)	
			canthin-6-one (**135**)	24.1 (K1); 9.1 (F32); 14.6 (PFB); 18.2 (FcB1)	42.7 (MCR5)	[65]
			5-methoxycanthin-6-one (**136**)	20.4 (K1); 41.6 (F32)	–	
Simaroubaceae	*Eurycoma longifolia*	CH_2_Cl_2_	eurycomanone (**137**)	0.06 (D6); 0.04 (W2)	0.02 (A-549); <0.006 (MCF-7)	[66,67]
			pasakbumin B (**138**)	0.08 (D6); 0.05 (W2)	0.02 (A-549); <0.006 (MCF-7)	
	*Picrolemma sprucei*	Hexane/H_2_O	neosergeolide (**139**)	0.002 (K1)	–	[68]
Apocynaceae	*Aspidosperma vargasii*	EtOH	ellipticine (**140**)	0.07 (K1)	–	
	*A. desmanthum*	EtOH	aspidocarpine (**141**)	0.02 (K1)	–	
Piperaceae	*Pothomorphe peltata*	CHCl_3_/EtOH	4-nerolidylcatechol (**142**)	0.7 (K1)	–	
Theaceae	*Camellia sinensis*		mefloquine (**143**)	–	–	[69]
			gallocatecin (**144**)	–	–	
Tiliaceae	*Grewia bilamellata*	MeOH	3α,20-lupandiol (**145**)	19.8 (D6); 19.1 (W2)	>90 (KB)	[29]
			grewin (**146**)	11.2 (D6); 5.5 (W2)	>107.5 (KB)	
			nitidanin (**147**)	21.2 (D6); 18.4 (W2)	>90 (KB)	
			2α,3β-dihydroxyolean-12-en-28-oic acid (**148**)	21.1 (D6); 8.6 (W2)	51.5 (KB)	
			2,6-dimethoxy-1-acetonylquinol (**149**)	42.2 (D6); 23.0 (W2)	169 (KB)	
Verbenaceae	*Lippia javanica*	EtOAc (aerial parts)	lippialactone (**150**)	23.8 (D10)	–	[70]

^a^ IC_50_: Concentration that resulted in 50% death of *Plasmodium falciparum*. ^b^ ED_50_: Concentration that resulted in 50% cell death.

**Table 3:** The ethnology of plants.

Family	Ethnologic Plant	Country	Plant Part	Antiplasmodial Activity (IC_50_) (μg/mL, Unless Indicated) ^a^ (*P. falciparum*)	Cytotoxicity (CC_50_ for Cells, LD_50_ for Brine Shrimp) (μg/mL, Unless Indicated) ^b,c^ (Cell Line)	References
Acanthaceae	*Justilia schimperand* (Hochst ex Nees) T. Alnder		Roots	–	–	[71]
Anacardiaceae	*Mangifera indica* L.	Africa	Leaves	% parasitaemia reduced from 8.9 at 60 mg/kg to 7.2 at 240 mg/kg (mice)	208.3 mg/kg (mice)	[72]
		Nigeria	Leaves	–	3079.1 (brine shrimp)	[73]
		Nigeria	Stem barks	–	2456.0 (brine shrimp)	[73,74]
	*Pseudoprotorhus longifolius* H. Perr.	Madagascar	Leaves	–	–	[75]
	*Rhus taratana* (Bak.) H. Perr.	Madagascar	Leaves	–	–	[75]
	*Sclerocarya birrea* (A. Rich) Hochst.	South Africa	Stem-bark (MeOH)	5.91 (D6)	–	[76]
	*S. caffra* Sond.	Madagascar	Leaves	–	–	[75]
Annonaceae	*Annona senegalensis* Rolyns &Gh	Nigeria	Leaves	–	6811.0 (brine shrimp)	[73]
	*Enantia chlorantha* Oliv.	Nigeria	Stem barks	–	214.3 (brine shrimp)	[73,74]
Apocynaceae	*Alstonia boonei* DeWild	Nigeria	Leaves; stem barks	% parasitaemia reduced from 19.4% (negative control) to 5.5% at 240 mg/kg (mice)	78.77 mg/kg (mice)	[72,74]
	*Aspidosperma cylindrocarpon* Müll. Arg.	Brazil	Trunk woods (EtOH)	44.0 (W2); 39.0 (3D7)	>500 (Vero)	[7]
	*A. parvifolium* A. DC.	Brazil	Trunk barks (EtOH)	32.8 (W2); 20.5 (3D7)	>500 (Vero)	[7]
	*A. olivaceum* Müll. Arg.	Brazil	Leaves (CH_2_Cl_2_)	7.0 (W2); 25.5 (3D7)	>500 (Vero)	[7]
			Leaves (EtOH)	7.0 (W2); 5.0 (3D7)	–	
			Trunk wood (CH_2_Cl_2_)	<6 (W2); <6 (3D7)	>500 (Vero)	
			Trunk bark (CH_2_Cl_2_)	<6 (W2); <6 (3D7)	–	
			Trunk bark (EtOH)	5.0 (W2); 7.0 (3D7)	>500 (Vero)	
	*A. ramiflorum* Müll. Arg.	Brazil	Leaves (EtOH)	32.8 (W2); 20.5 (3D7)	–	[7]
			Leaves (CH_2_Cl_2_)	<6 (W2); <6 (3D7)	–	
			Trunk woods (EtOH)	36.5 (W2); 48.0 (3D7)	–	
			Trunk woods (CH_2_Cl_2_)	9.5 (3D7)	>500 (Vero)	
			Trunk woods (EtOH)	19.8 (W2); 1.0 (3D7)	–	
			Trunk barks (CH_2_Cl_2_)	<6 (W2); <6 (3D7)	>500 (Vero))	
	*A. spruceanum* Benth. ex Müll. Arg.	Brazil	Leaves (EtOH)	65.0 (W2); >100 (3D7)	–	[7]
			Leaves (CH_2_Cl_2_)	23.25 (W2); 47.0 (3D7)	–	
			Trunk woods (EtOH)	29.5 (W2); 41.5 (3D7)	–	
			Trunk woods (CH_2_Cl_2_)	<6 (W2); <6 (3D7)	109.6 (Vero))	
			Trunk woods (CHCl_3_)	37.0 (W2); >100 (3D7)	–	
			Trunk barks (EtOH)	26.3 (W2); 14.0 (3D7)	–	
			Trunk barks (CH_2_Cl_2_)	<6 (W2); <6 (3D7)	–	
			Trunk barks (EtOH)	28.0 (W2); 19.0 (3D7)	–	
	*A. tomentosum* Mart.	Brazil	Trunk woods (EtOH)	26.5 (W2); 25.0 (3D7)	–	[7]
			Leaves (EtOH)	23.8 (W2); 27.0 (3D7)	–	
			Fruits (EtOH)	20.5 (W2); 38.6 (3D7)	–	
			Seeds (EtOH)	24.5 (W2); 3.0 (3D7)	>500 (Vero))	
Aristolochiaceae	*Aristolochia acuminata* Lamk.	Madagascar	Roots, stems, leaves	–	–	[75]
Asteraceae	*Artemisia annua* L.	China	Whole plants	–	–	[77]
	*Tithonia diversifolia* A. Gray	Nigeria	Leaves	–	2304 (brine shrimp)	[73]
	*Vernonia amygdalina* Del.		Leaves	–	–	[71]
Avicenniaceae	*Avicennia marina* (Forsk) Vierh.	Madagascar	Aerial parts	–	–	[78]
	*A. basilicum* L.	Madagascar	Aerial parts	–	–	[75]
Bignoniaceae	*Fernandoa* sp.	Madagascar	Aerial parts	–	–	[75]
	*Kigelianthe madagascariensis* Sprague var. hidebrandtii	Madagascar	Leaves	–	–	[75]
Brassicaceae	*Brassica nigra* (L.) Koch.		Seeds	–	–	[71]
Caricaceae	*Carica papaya* L.		Leaves, fruits, roots		–	[71,79]
Celastraceae	*Maytenus acuminata* (L.f.) Loes	Kenya	leaves, root barks	36.6–41.5%	–	[80]
Combretaceae	*Combretu raimbaulti* Heckel	Madagascar	Leaves		–	[75]
	*Terminalia catappa*	Nigeria	Leaves (EtOAc)	3.1 (K1)	159.9 μg/L (L6)	[81]
	*T. latifolia* Engl.	Nigeria	leaves	–	272.9 (brine shrimp)	[73]
Commelinaceae	*Commelina benghalensis* L.	Madagascar	Aerial parts	–	–	[75]
Compositae	*Brachylaena ramiflora* (DC.) H. Humb	Madagascar	Aerial parts	–	–	[75]
	*Conyza aegytiaca* Ait. Var *lineariloba*	Madagascar	Aerial parts	–	–	[75]
	*Inula perrieri* H. Humb.	Madagascar	Leaves	–	–	[75]
	*Parthenium hysterophorus* L.	Madagascar	Aerial parts	–	–	[75]
	*Senecio ompricaefolius* (ex DC.) H. Humb.	Madagascar	Aerial parts	–	–	[75]
	*Stenocline inuloides* DC.	Madagascar	Leaves	–	–	[75]
	*Tagetes erecta* L.	Madagascar	Leaves	–	–	[75]
	*T. patula* L.	Madagascar	Leaves	–	–	[75]
	*Vernonia lasiopus* O. Hoffm.	Kenya	Root barks	–	–	[75]
	*V. pectoralis* Bak.	Madagascar	Aerial parts	–	–	[75]
	*V. trichodesma* Bak.	Madagascar	Leaves	–	–	[75]
	*V. chapelieri* Drak.	Madagascar	Aerial parts	–	–	[75]
	*V.* sp. (Dr. Hely)	Madagascar	Aerial parts	–	–	[75]
	*V. ampandrandavensis* Bak.	Madagascar	Aerial parts	–	–	[75]
Cucurbitaceae	*Momordica charantia* L.	Madagascar	Aerial parts	–	–	[75]
	*Zehneria scabra* (Lf.) Sond.		Roots	–	–	[71]
Euphorbiaceae	*Bridelia micrantha* Benth.	Nigeria	Leaves	–	>90,000 (brine shrimp)	[73]
	*Croton goudoti* H. Bn.	Madagascar	Leaves	–	–	[75]
	*C. macrostachyus* Hochst. Ex Del.		Leaves/barks/roots	–	–	[71]
	*Euphorbia hirta*	Nigeria	Whole plants (Hexane)	4.3 (K1)	14.2 (L6)	[81,82]
	*Flueggea microcarpa* Blume	Madagascar	Aerial parts		–	[75]
	*Jatropha curcas* L.	Nigeria	Leaves (EtOAc)	2.4 (K1)	126.5 (L6)	[75,81,82]
		Madagascar	leaves, roots			
	*Manihot utilisma* Pohl.	Madagascar	Leaves	–	–	[75]
	*Phyllanthus amarus* Schum. & Thonn.	Brazil, Cuba, Haiti, Nigeria, Elsewhere	Whole plants (MeOH)	5.0 (3D7)	–	[83,84]
			Whole plants (CH_2_Cl_2_)	14.5 (3D7)	–	
		India	Whole plants	–	–	[85]
		Nigeria	Leaves (EtOAc)	5.6 (K1)	77.7 (L6)	[81,82]
		Ghana	Whole plants	–	–	[85]
		West Africa	Aerial parts	–	–	
	*Phyllanthus* sp.	Madagascar	Aerial parts	–	–	[75]
Fabaceae	*Acacia nilotica* L.	Pakistan	Leaves (EtOH)	1.3 (3D7)	–	[86]
	*Caesalpinia benthamiana*	Guinea	Leaves (MeOH)	4.0 (Ghana)	32.0 (MRC-5)	[79]
	*Cajanus cajan* Mill sp.	Nigeria	Leaves	–	988.5 (brine shrimp)	[73,74]
	*Calliandra haematocephala* Hassk	Nigeria	Roots	–	–	
	*Calpurna ourea* (Ait.) Benth		Leaves	–	–	[71]
	*Cassia siamea*	Nigeria	Stem barks (EtOAc)	2.70 (K1)	988.5 (stem bark), 8232.2 (brine shrimp)	[73]
			leaves	–		
	*Piliostigma thonnigii* Schum	Nigeria	Leaves	–	7958.0 (brine shrimp)	[73]
Flacourtiaceae	*Homalium* sp.	Madagascar	Aerial parts	–	–	[75]
Gramineae	*Phragmites mauritianus* Kunth	Madagascar	Aerial parts	–	–	[75]
Hydrengeaceae	*Dichroa febrifuga*	China	Roots	–	–	[87]
Icacinaceae	*Cassinopsis madagascariensis* (Baill.) H. Bn.	Madagascar	Leaves, stem barks	–	–	[75]
Lamiaceae	*Hyptispectinata* Poit.	Madagascar	Leaves	–	–	[75]
	*Ocimum canum* Sims.	Nigeria	Leaves (EtOAc)	1.8 (K1)	60.1 (L6)	[75,81]
		Madagascar	Stems, seeds	–		
	*O. lamiifolium* Hochst. ex Benth.		Leaves	–	–	[71]
	*Cassytha filiformis* L.	Nigeria	Vines	–	–	[74]
	*Cinnamomum camphora* (L.) Sieb	Madagascar	Leaves	–	–	[75]
Leguminosae	*Abrus precatorius* L.	Madagascar	Leaves	–	–	[75]
	*Albizzia lebbek* Benth.	Madagascar	Aerial parts	–	–	[75]
	*Caesalpinia bonducella* Fleming	Madagascar	Seeds, roots	–	–	[75]
	*Cassia occidentalis* L.	Madagascar	Aerial parts	–	–	[75]
	*Crotalaria spinosa* Hochst.	Madagascar	Leaves	–	–	[75]
	*Erythryna indica* Lamk.	Madagascar	Aerial parts	–	–	[75]
	*Piliostigma thonningii*	Nigeria	Leaves (EtOAc)	3.6 (K1)	56.1 (L6)	[81]
	*Pongamia pinnata* L.	India	Barks (MeOH)	11.7 (CQ-sensitive)	>200 (THP-1)	[88]
Lilliaceae	*Allium sativum* L.		Bulbs	–	–	[71]
Loganiaceae	*Anthocleista amplexicaulus* Bak.	Madagascar	Aerial parts	–	–	[75]
	*A. rhizophoroides* Bak.	Madagascar	Roots, leaves	–	–	[75]
	*Strychnos mostuoides* Leeuwenberg	Madagascar	Aerial parts	–	–	[75]
Malvaceae	*Gossypium arboreum* L.	Nigeria	Leaves	–	94.1 (brine shrimp)	[73]
	*G. barbadense* L.	Nigeria	Leaves	–	3585.0 (brine shrimp)	[73]
	*G. hirsitum* L.	Nigeria	Leaves	–	257.2 (brine shrimp)	[73]
Meliaceae	*Azadirachta indica* A. Juss	Africa	leaves	The percentage parasitaemia reduced from 15.7 % to 4.8 % at 240 mg/kg (in vivo)	140.0 mg/kg (mice)	[72]
	*Swietenia macrophylla* King	Indonesia	Seeds	–	–	[89]
			Barks	78% inhibition at 100 (Indo)	–	[90]
Melianthaceae	*Bersama abyssinica* Fresen.		Leaves, root barks and stems	–	–	[71]
Menispermaceae	*Burasaia australis* Sc. Elliot	Madagascar	Root barks	–	–	[75]
	*B. congesta* Decne	Madagascar	Root barks	–	–	[75]
	*B. gracilis* Decne	Madagascar	Root barks	–	–	[75]
	*Burasaia madagascariensis* Thou.	Madagascar	Root barks	–	–	[75]
	*B. nigrescens* R. Cap.	Madagascar	Root barks	–	–	[75]
	*Chasmanthera uviformis* Baill.	Madagascar	Stem barks	–	–	[75]
	*Cissampelos pareira* L.	Madagascar	Roots	–	–	[75]
	*C. madagascariensis* (Baill.) Diels.	Madagascar	Roots	–	–	[75]
	*Spirospermum penduliflorum* Thou.	Madagascar	Roots, stem barks	–	–	[75]
	*Strychnopsis thouarsii* Baill.	Madagascar	Leaves, root barks	–	–	[75]
	*Triclisia macrocarpa* (Baill.) Diels	Madagascar	Root barks, stem barks	–	–	[71]
Mimosaceae	*Acacia catechu* (L.f.) Willd.		Leaves	–	–	[71]
Moraceae	*Ficus elastica* Roxb. ex Hornem.	Cameroon	Roots (MeOH)	9.5	–	[91]
	*F. sur* Forssk.	Kenya	Leaves, stem barks, root barks	34.1–48.4% Inhibition	–	[80]
	*F. thonningii* Blume	Nigeria	Leaves (Hexane)	2.7 (NF54); 10.4 (K1)	>20 (KB)	[90]
Myrtaceae	*Psidium guajava* L.	Nigeria	Stem barks	–	707.2 (brine shrimp)	[72]
Ochnaceae	*Lophira alata* Banks	Nigeria	Leaves (Hexane)	2.5 (NF54); 2.5 (K1)	>20 (KB)	[90]
Papilionaceae	*Pericopsis elata* Harms	Nigeria	leaves	–	601.8 (brine shrimp)	[73]
	*Pterocarpus osun* Craib	Nigeria	Stem barks	–	–	[74]
Periplocaceae	*Cryptolepts sanguinolenta*	West Africa	Roots	–	13.9 (MCF7)	[92]
	*Parquetina nigrescens* (Afz.) Bullock	Nigeria	Root barks	–	–	[74]
Phytolaccacaa	*Phytolacca dodecandra* L’Hér.		Leaves	–	–	[71]
Polygonaceae	*Rumex abyssinicus* Jacq.		Leaves and stems	–	–	[71]
Potamogetonaceae	*Potamogeton javanicus* Hass Karl	Madagascar	Aerial parts	–	–	[75]
Ranunculaceae	*Clematis mauritiana* Lamk. Var. *normalis*	Madagascar	Aerial parts	–	–	[75]
Rhamnaceae	*Rhamnus prinoides* L’ H′erit	Kenya	Leaves, root barks	34.1–43.9% Inhibition	–	[80]
	*R. staddo* A. Rich.	Kenya	Root barks	11.1% Inhibition	–	[80]
Rubiaceae	*Anthospermum emirnense* Bak.	Madagascar	Aerial parts	–	–	[75]
	*Cinchona ledgeriana* Muens	Madagascar	Stem barks	–	–	[75]
	*C. offlcinalis* L.	Madagascar	Stem barks	–	–	[75]
	*C. succirubra* Pavon et Kiutzsch	Madagascar	Stem barks	–	–	[75]
	*Cephalanthus spathelliferus* Bak.	Madagascar	Leaves	–	–	[75]
	*Danais fragrans* Gaertn.	Madagascar	Roots	–	–	[75]
	*D. gerrardii* Bak.	Madagascar	Roots	–	–	[75]
	*D. verticillata* Bak.	Madagascar	Roots	–	–	[75]
	*D. breviflora* Bak.	Madagascar	Roots	–	–	[75]
	*D. cernua* Bak.	Madagascar	Roots	–	–	[75]
	*Hymenodyction lohavato* baill.	Madagascar	Root barks, stem barks	–	–	[75]
	*Morinda lucida* Benth	Africa	Leaves	The percentage parasitaemia reduced from 14.0 % to 5.8 % at 240 mg/kg (in vivo)	134.5 mg/kg (mice)	[72]
		Nigeria	Stem barks	*P. berghei*	2.6 (brine shrimp)	[73]
		Nigeria	Leaves	–	383.9 (brine shrimp)	[73]
	*Nauclea latifolia* S.M.	Nigeria	Stem barks	–	9368.0 (brine shrimp)	[73]
	*Saldinia* sp. (andriambavifoy)	Madagascar	Aerial part	–	–	[75]
	*Sarcocephalus latifolius* (J. E. Smith) E. A. Bruce	Nigeria	Root barks	–	–	[74]
	*Schismatoclada concinna* Bak.	Madagascar	Root barks	–	–	[75]
	*S. farahimpensis* Bak.	Madagascar	Root barks	–	–	[75]
	*S. viburnoides* Bak.	Madagascar	Root barks	–	–	[75]
	*Citropsis articulata* (Willd. ex Spreng.) Swingle & Kellerman	Uganda	Roots	77% inhibition at 10 (FcB1)	12% inhibition at 10 (Vero)	[64]
			Demethylsuberosin	16.7	>50% inhibition at 16.7 (Vero)	
			5-hydroxynoracronycine	0.9	9.3% inhibition at 0.9 (Vero)	
			1,5-dihydroxy-2,3-dimethoxy-10-methyl-9-acridone	3.0	30.5% inhibition at 3.0 (Vero)	
			7α-obacunyl acetate	9.3	>50% inhibition at 9.3 (Vero)	
Rutaceae	*Evodia fatraina* H. Perr	Madagascar	Root barks, stem barks	–	–	[75]
	*Toddalia asiatica* (L.) Lam.	Kenya; Madagascar	Root barks; root barks, stem barks	–	–	[75,80]
	*Zanthoxylum tsihanimpotsa* H. Perr.	Madagascar	Stem barks	–	–	[75]
Santalaceae	*Okoubaka aubrevillei* Phelleg & Nomand	Nigeria	Stem barks	–	–	[74]
Sapindaceae	*Dodonaea viscosa* Jacq.	Madagascar	Leaves	–	–	[75]
	*D. madagascariensis* Rdlk.	Madagascar	Leaves	–	–	[75]
Selaginellaceae	*Salaginella vogelli*	Cameroon	Leaves (MeOH)	32.2	–	[91]
Schizaeaceae	*Mohria caffrorum* (L.) Desv.	Madagascar	Aerial parts	–	–	[75]
Simaroubaceae	*Brucea antidysenterica* J.F. Mill.		Stems, barks seeds	–	–	[71]
Ulmaceae	*Trema commersonii* Boj.	Madagascar	Aerial part	–	–	[75]
	*T. orientalis* Blume	Madagascar	Root barks	2.0 (K1)	32.5 (L6)	[75]
Verbanaceae	*Lippia multiflora* Moldenke	Nigeria	Aerial part	–	1.1 (brine shrimp)	[73]
	*Clerodendrum myricoides* (Hochst.) Vatke	Kenya	Root barks	9.8% (*Plasmodium berghei* NK65)	–	[71,80]
	*Vitex doniana*	Nigeria	Leaves (Hexane)	3.6 (K1)	431.4	[81,82]
			Stem barks (Hexane)	6.8 (K1)	ND	[81]
Zingiberaceae	*Curcuma longa* L.	Madagascar	Leaves	–	–	[75]
	*Zingiber officinale* Roscoe		Rhizome	–	–	[71]

^a^ IC_50_: Concentration that resulted in 50% death of *Plasmodium falciparum*. ^b^ LD_50_: Concentration that was lethal to 50% of test animals. ^c^ CC_50_: Concentration that resulted in 50% cell death.
